# Broiler welfare trade-off: A semi-quantitative welfare assessment for optimised welfare improvement based on an expert survey

**DOI:** 10.1371/journal.pone.0222955

**Published:** 2019-10-01

**Authors:** Marc B. M. Bracke, Paul Koene, Inma Estevez, Andy Butterworth, Ingrid C. de Jong

**Affiliations:** 1 Wageningen Livestock Research, Wageningen University & Research, Wageningen, The Netherlands; 2 Neiker-Tecnalia Basque Institute for Agricultural Research and Development, Vitoria-Gasteiz, Spain; 3 Ikerbasque, Basque Foundation for Science, Bilbao, Spain; 4 School of Veterinary Sciences, University of Bristol, Bristol, England, United Kingdom; Tokat Gaziosmanpasa University, TURKEY

## Abstract

In order to support decision making on how to most effectively improve broiler welfare an innovative expert survey was conducted based on principles derived from semantic modelling. Twenty-seven experts, mainly broiler welfare scientists (n = 20; and 7 veterinarians), responded (response rate 38%) by giving welfare scores (GWS, scale 0–10) to 14 benchmarking housing systems (HSs), and explaining these overall scores by selecting, weighing and scoring main welfare parameters, including both input and output measures. Data exploration followed by REML (Linear Mixed Model) and ALM (Automatic Linear Modelling) analyses revealed 6 clusters of HSs, sorted from high to low welfare, i.e. mean GWS (with superscripts indicating significant differences): 1. (semi-natural backyard) Flock (8.8^a^); 2. Nature (7.7^ab^), Label Rouge II (7.4^ab^), Free range EU (7.2^ab^), Better Life (7.2^ab^); 3. Organic EU (7.0^bc^), Freedom Food (6.2^bc^); 4. Organic US (5.8^bcd^), Concepts NL (5.6^abcdef^), GAP 2 (4.9^bcd^); 5. Conventional EU (3.7^de^), Conventional US (2.9^ef^), Modern cage (2.9^abcdef^); 6. Battery cage (1.3^f^). Mean weighting factors (WF, scale 0–10) of frequently (n> = 15) scored parameters were: Lameness (8.8), Health status (8.6), Litter (8.3), Density (8.2), Air quality (8.1), Breed (8.0), Enrichment (7.0) and Outdoor (6.6). These did not differ significantly, and did not have much added value in explaining GWS. Effects of Role (Scientist/Vet), Gender (M/F) and Region (EU/non-EU) did not significantly affect GWS or WF, except that women provided higher WF than men (7.2 vs 6.4, p<0.001). The contribution of welfare components to overall welfare has been quantified in two ways: a) using the beta-coefficients of statistical regression (ALM) analyses, and b) using a semantic-modelling type (weighted average) calculation of overall scores (CalcWS) from parameter level scores (PLS) and WF. GWS and CalcWS were highly correlated (R = ~0.85). CalcWS identified Lameness, Health status, Density, Breed, Air quality and Litter as main parameters contributing to welfare. ALM showed that the main parameters which significantly explained the variance in GWS based on all PLS, were the output parameter Health status (with a beta-coefficient of 0.38), and the input parameters (stocking) Density (0.42), Litter (0.14) and Enrichment (0.27). The beta-coefficients indicated how much GWS would improve from 1 unit improvement in PLS for each parameter, thus the potential impact on GWS ranged from 1.4 welfare points for Litter to 4.2 points for Density. When all parameters were included, 81% of the variance in GWS was explained (77% for inputs alone; 39% for outputs alone). From this, it appears that experts use both input and output parameters to explain overall welfare, and that both are important. The major conventional systems and modern cages for broilers received low welfare scores (2.9–3.7), well below scores that may be considered acceptable (5.5). Also, several alternatives like GAP 2 (4.9), Concepts NL (5.6), Organic US (5.8) and Freedom Food (6.2) are unacceptable, or at risk of being unacceptable due to individual variation between experts and farms. Thus, this expert survey provides a preliminary semi-quantified decision-support tool to help determine how to most effectively improve broiler welfare in a wide range of HSs.

## Introduction

Ideally, efforts to address positive changes in animal welfare should follow the principles underlying effective altruism [1, 2], namely to maximise efficiency in doing good. Setting priorities with this in mind, however, is not always an easy task, because, overall, welfare is difficult to quantify, and an animal’s welfare can be affected by many factors.

In the past, a semantic modelling methodology has been developed to assess overall welfare, expressed as a score on a scale from 0 to 10, and used to prioritize welfare aspects in a systematic, formalised and science-based way [3, 4]. This methodology, which involved empirical as well as expert validation surveys, has been applied to various farmed species, including pigs (Sow WELfare Model (SOWEL), [5], PIGTAIL on tail biting [6, 7] and RICHPIG on pig enrichment [8]), laying hens [9], dairy cattle [10], calves [11]) and farmed salmon [12–14]. The FOWEL (Fowl WELfare) model for laying hens was previously used in decision making in the field of effective altruism, but its validity has been questioned [15, 16]. To date, no semantic model has been developed to assess the welfare of broiler chickens, and welfare scores for (attributes of) housing systems are lacking.

Broiler welfare is an issue of concern. Various alternative production systems have been implemented to improve broiler welfare, e.g. organic production in the EU [17], Label Rouge in France [18] and Better Life (Beter Leven) in the Netherlands [19]). Although it is generally recognised that system properties such as stocking density, litter quality and the selection for efficient growth have contributed to broiler welfare problems [18, 20, 21], the relative contribution of the various welfare measures to overall welfare is not clear. Some publications have started addressing the issue, such as Gocsik et al. [22] who used scores for Welfare-Quality measures to estimate the costs of welfare improvements in a small number of production systems. Using the full Welfare-Quality protocol, however, most conventional farms were found to be ‘acceptable’ [23], and it remains to be seen if the suggestion that most conventional farms are ‘acceptable’, is in accordance with expert opinion. Hence, a systematic, semantic-modelling type quantification seemed called for to assess welfare in a wider range of systems for the purpose of benchmarking, and to unravel the contribution of the various welfare measures to the overall welfare of broiler chickens. Therefore, we conducted an expert elicitation to provide a science-based and (semi-) quantified decision-support tool with the aim to ultimately help stakeholders, such as chain actors, policy makers and NGOs, make value-for-money decisions regarding potential improvements in broiler welfare. The expert elicitation involved asking established welfare scientists and others (e.g. veterinarians) to provide welfare scores and weighting factors for welfare-relevant attributes/parameters of broiler housing systems. The survey was international, with a focus on regions including the US and the EU. The method aimed to especially compare conventional systems with alternative systems, and also to compare with potential ‘control’ systems, namely a semi-natural backyard flock and living under natural conditions (positive controls), and a battery-cage type system for rearing broilers (negative control). In particular, the objective was to answer the question: What are the factors which affect the welfare of broilers, and what is their relative importance and contribution to overall welfare in a specified selection of benchmark systems? To this end, we report an international expert survey, with at its core, a cross-table listing 14 conventional and alternative broiler housing-and-management systems (HSs) with their overall given welfare scores (GWS), the main welfare parameters with their relative importance scores, called weighting factors (WF), and welfare-component scores, called parameter level scores (PLS), together expressing how the latter (PLS and WF) relate to GWS for the benchmark HSs.

## Materials and methods

### Background and terminology

A semantic-modelling type expert elicitation was set up to allow the respondents to explain GWS (see S1 File ‘Survey, invitation and background’). The process allowed linking of the experts’ scientific knowledge, either explicitly or implicitly, to welfare (GWS) in a parsimonious and systematic way. Overall welfare was (re-)calculated as a weighted average of component (PLS and WF) scores [3]. Welfare components are referred to as ‘parameters’, formerly labelled as ‘attributes’ of HSs [3, 5] or ‘welfare indicators’ [12]. Parameters are welfare-relevant properties of a housing-and-management system (HS), where a HS does not just refer to the physical environment (building), but also to the animals (social environment) and the people in the system. Thus, a welfare parameter may be an input, or an output, which is *grosso modo* equivalent to environment-based, or animal-based, also referred to as design-based, or welfare-performance-based measure. In Anonymous [24] and Bracke [8] the underlying framework for this methodology and terminology have been described in more detail, and in these previous studies we used related, but not identical terms. Most, but not all, animal-based parameters are output or welfare-outcome-based. For example, breed is an animal-based parameter that is an input, rather than an output as regards the assessment of animal welfare, which can be defined as “what matters to the animals from their points of view” [25]. Both input and output parameters were included in the survey in the current study because some scientists differ almost fundamentally regarding the issue of which types of parameters should be used for welfare assessment, for example that only animal-based output parameters are valid in principle [26] or that it is essential to use a mix of input and output measures [27].

### Expert survey (and non-statistical results)

#### Expert selection and communication

An expert survey was conducted via email in April 2018. An invitation (see S1 File) was sent to an initial set of 35 experts, selected on the basis of recommendations by 3 key experts and a scan of the broiler-welfare assessment literature. In the invitation, experts were asked whether they were willing to share their views on integrated welfare assessment of chickens kept for meat production, assessed at the housing and management ‘systems’-level. The experts were asked to provide overall welfare scores for 5–6 typical HSs/farms, WF and PLS scores for 4 or 5 main welfare parameters, thus tentatively expressing what kind of welfare improvement might be expected by adopting feasible alternative systems. Shopping vouchers were promised for completed surveys. When individuals were unable to participate, alternative names were requested. This approach was intended to include a full range of generalist and senior scientists explicitly knowledgeable in the area of broiler welfare.

#### Survey

Two versions of the survey were available, a primary version in Microsoft Excel and a backup Word version (see S1 File). The Excel version had checks built-in for maintaining data integrity. The Excel template also generated 3 bar charts showing how PLS, with or without WF, related to GWS in various ways (weighted and unweighted and with/without rescaling, see the figures below). These charts enabled the experts to visually check their own scoring consistency.

The survey presented 16 specific requests, as well as 6 colour-coded tables, which are referred to in this paper by their numbering in the survey as shown in S1 File.

Table A in S1 File showed a pre-defined list of HSs and a column to assign GWS. Table B in S1 File allowed for the description of new or modified HSs, preferably after checking Table C in S1 File which provided more detailed descriptions of the HSs listed in Table A in S1 File. Table D in S1 File took the HS labels and GWS from Table A in S1 File as column headings to produce a cross-table to assign PLS for parameters and their WF taken from Table E in S1 File. Tables F and G in S1 File provided more detailed descriptions of the parameters listed in Table E in S1 File, and allowed for the respondent to modify the description and/or add new parameters to the list. Experts were allowed to select their own HSs and parameters, because reliable scores required sufficient familiarity with the system and parameters. Experts were requested to focus on the 4 or 5 main parameters, because it was considered to be quite demanding of the respondents to provide more PLS.

#### Housing systems

When selecting HSs from Table A in S1 File, experts were requested: (1) to cover the whole scale by including at least one low-welfare HS (GWS< 3) and one high-welfare HS (GWS>7); (2) to focus on 'average farms'; (3) to focus on well-known systems; and (4) on feasible alternatives. Alternative HSs included schemes defined by farmers (NCC [28]), retailers, food chains and governmental bodies (organic farming in the EU [17]), and HSs developed by NGOs (Freedom Food in the UK and Better Life in the Netherlands [19]). In total, the experts were asked to assess 5–6 HSs: 1 conventional, 2–3 alternatives, 1 high, and 1 low welfare HS. The following pre-defined HSs were marked * indicating that they were preferred for scoring: Battery cage*, Conventional US*, Conventional EU*, Organic US*, Organic EU* and Flock*.

Tables A and B in S1 File (not shown here, see the survey in S1 File) allowed the respondent to select, add, score and modify HS descriptions detailed in Table 1 (which was Table C in S1 File).

**Table 1:** (Table C in S1 File). Broiler housing-and-management system (HS) labels with more extended descriptions. Note: The table gives indicative HS descriptions only (which the expert respondent was allowed to modify).

HS label	HS description
Conventional US*	Average/typical conventional farm in the US*; ~20.000ft or 1860m^2^/house; 32-43g/m^2^; 6.5-9lbs/ft^2^; ~22.000birds/house; fast-growing (Cobb): 58g/d; ~2.7kg BW in ~46d; wood shavings;1x/day checked; litter reused in multiple rounds; natural/tunnel ventilation; no enrichment (besides litter); no outdoor; dim light; controlled light, typically 23:1 L:D day 1–7, >day 7: 20:4 L:D; ~4.5% mortality; no welfare legislation.
Conventional EU*	Average/typical conventional farm in the EU*; ~22birds/m^2^;~33-42kg/m^2^; 6.8–8.5lbs/ft^2^; ~10–20.000birds/house; fast-growing: ~55–60.5g/d; ~2.3kg in 35-40d; woodshavings-based litter on concrete; no enrichment (besides litter); no outdoor; severe pododermatitis: 20–30%; thermal control; thinning; 6h/d dark (4h continuous; EC Directive); fully indoors; ~3–5% mortality.
Battery cage*	Average/typical (traditional) battery cage for broilers*; ~45kg/m^2^; ~30birds/m^2^ @1.5kg; ~400cm^2^/bird; groups of ~6 birds/cage; fast growing; metal wire floor & cage; no enrichment; no outdoor.
Modern cage	Modern colony cage (nylon wire floor above manure belt, in-house hatching, automated harvesting); ~18birds/m^2^; ~45kg/m^2^; up to 4 tiers; colony of ~100 birds/cage; ~25.000 birds/house; fast-growing: ~64g/d; ~2.7kg BW in~40d; no enrichment; no outdoor; thermal controlled building; 2–6% mortality.
Organic US*	Average/typical organic farm in the US*; not regulated; outdoor may be veranda/porch/moveable coop; no specific welfare requirements (only organic food & treatment).
Organic EU*	Average organic farm in the EU*; <1600m^2^/enclosure; fixed housing (≤10birds/m^2^ & 21kg/m^2^) & ≥4 m^2^/bird outdoors; <4.800 birds per enclosure; slow-growing: ≥81d; 2.6kg BW in 70d, ~37g/d (NL); >1/3 solid floor with litter e.g. straw/wood shavings; enrichment grain or straw; ≥1/3 of life outdoor; mainly covered by vegetation & cover & easy access to food & water outdoors; roughage or silage provided daily; severe FPD ~60%; natural ventilation; perches; ≥8 h continuous dark; mortality 3%; EU regulations enforced (~1x/yr unannounced visit); immediate, allopathic treatment, records kept.
Flock*	Small backyard flock, with mother hen & cock, extensive semi-natural environment, quality housing & (health) care*; stable family group, ~10 adult birds/10.000m^2^ backyard with vegetation & cover; local breed; balanced diet; protected from predators.
Nature	Wild jungle fowl living in a fully natural environment.
Freedom Food	Freedom food/RSPCA assured (average farm); 30kg/m^2^; 19birds/m^2^; ≤10.000birds/enclosure; no limit regarding strain or slaughter age; some enrichment (besides litter); no outdoor; 1 nipple/10birds, 1 cup/28birds; 25mm linear or 16mm circular feederspace/bird; >8h light; >20lux; ≥6h continuous dark/d; 14h light >5d.
Better Life	Better Life scheme of Dutch Society for Protection of Animals (average farm); ~25-30kg/m^2^; 12birds/m^2^+167cm^2^/bird covered veranda/porch (≥20% of total area; 35d used); large group; slower growing: 45g/d; 2.3kg BW in ~56d; concrete & woodshavings; enrichment: 2g grain spread (from day 15) or 1 strawbale/1000birds; 100% plant-based, ≥70% grains; severe footpad dermatitis ~3%; 8h/d dark; ≥20lux natural light; mortality: ~3%.
Concepts NL	Dutch retailer scheme(s) (e.g. AH; 'middle segment' between conventional & Better Life); ~33-38kg/m^2^; 14-19birds/m^2^; large group; slower growing: 49g/d; 2.4kg BW in ~46d; e.g. Hubbard JA 987; concrete, woodshavings; enrichment: 2g grain spread (as of day 15); 1 strawbale/1000birds (or 1 pecking stone/200m); no outdoor; 6h/d dark; daylight; ~2.5% mortality.
Free range EU	Average/typical free-range farm in the EU; 19birds/m^2^ or 27.5kg/m^2^ indoor & 1m^2^/bird outdoor (i.e. average 0.95bird/m^2^); 2.1kg BW in 56d; enrichment grain or straw; 50% of life outdoor access; ≥70g grain in feed; severe FPD ~3–5%; daylight; 8h dark; mortality: ~2.5%.
Label Rouge II	Label Rouge type II (average farm); 20 birds/m^2^+outdoor (2m^2^/bird) (i.e. average 0.5 bird/m^2^, ~1kg/m^2^); ≤1.000birds/enclosure; slow growing (e.g. Hubbard): >81d; selected for breeding & body composition (breasts, low fat, thin skin); no animal food, >75% cereals.
GAP 2	Global Animal Partnership step 2; ~32kg/m^2^; large group; fast-growing: 58g/d; 2.7kg BW in 46d; woodshavings; 1 enrichment (e.g. straw bale)/70m^2^; no outdoor; 1% of total diet grain in feed; 8h/d dark; some natural light via ventilators.

*: Preferred HSs for scoring by assigning a given welfare score (GWS) on a scale from 0 to 10. ~: about; BW: Body weight; L:D: Light: dark.

The HSs were derived from the literature. An example; searching for the terms ‘Freedom Food’ and ‘Label Rouge’ in De Jong et al. [21] and SCAHAW [18]; and for HS descriptions in Ellen et al. [19], Gocsik et al. [22], and Vissers [29]. The latter described HSs for broilers in the USA and the Netherlands.

The list of HSs contained one potentially poor-welfare system, namely Battery cage* (for rearing broilers), and two potentially high-welfare systems (Flock* and Nature), which were not currently feasible for commercial-scale broiler production. This left 9 more or less feasible alternative systems to be compared to conventional: 2 organic systems (US and EU), 2 NGO-based welfare schemes (Freedom Food and Better Life), three legislated schemes in the EU (Organic EU*, Free range EU and Label Rouge II), and two retailer-originated schemes (Concepts NL and GAP 2). Alternative HSs included systems from the Netherlands (Concepts NL and Better Life), France (Label Rouge II), the US (GAP 2) and the UK (Freedom Food).

#### Parameter weighting

Experts were asked to give WF scores, i.e. relative importance scores on a scale from 0–10, for 4–5 main parameters selected from Table E in S1 File (Table 2). Scoring other, less important parameters was optional, and some parameters were marked * indicating that they were preferred for assigning WF (Space/pen*, Density*, Breed*, Litter*, Outdoor*). The parameter list was based on the pilot interviews, literature and points of concern frequently mentioned by NGOs [30]. In a separate table (Table F in the survey, not shown here, see S1 File) parameters could be (re)defined and weightings could be explained.

**Table 2:** (Table E in S1 File). List of parameters. In this table respondents were requested to select and score the 4 or 5 main parameters by assigning a weighting factor (WF), expressing relative importance for welfare assessment on a scale from 0 to 10. New parameters could also be added.

WF	Parameter label	Parameter description (may alter yellow cells below)
	Space/pen*	Space/pen (m^2^); total enclosure size (indoor & outdoor)
	Density*	Stocking density (e.g. kg/m^2^)
	Group size	Social contact (group size)
	Breed*	Breed (esp. growth rate) (& other type-of-bird-related characteristics)
	Litter*	Floor quality (presence of litter; litter quality)
	Air quality	Air quality (fresh air, dust, NH_3_, humidity)
	Enrichment	Enrichment/stimulation, e.g. straw bales, strings, platforms, etc.
	Outdoor*	Outdoor access & quality of outdoor area
	Foraging	Ability to search for food, e.g. scratching for grains in litter
	Water	Water quantity, quality, drinker type
	Fd level (E)	Feeding energy level/schedule/system
	Fd quality	Food quality nutritionally (other than energy) & food hygiene
	Dust bath	(Quality of) dustbathing ability
	Fd selection	Ability to select ingested food items, e.g. variation in food items & palatability
	Lameness	Lameness & other locomotion problems
	Skin&plumage	Skin problems & plumage condition
	Heat	Exposure to heat stress & general thermal regulation
	Cold	Exposure to cold stress
	Fd competition	Competition for food
	Group stability	Social stability (e.g. mixing)
	Handling	Handling & other issues related to fear of humans
	Disturbance	Disturbance e.g. predation/panic (non-human/social)
	Cover	Cover to hide (e.g. for predators & conspecifics)
	Moveability	Ability to move around & movement comfort
	Mother/family	Mother hen presence, family group, group composition
	Find fd&water	Ability to learn to find food & water at an early age
	Perch/rest	Perching & resting comfort (presence, perch quality, length)
	Preen/comfB	Preen/comfort behaviour (e.g. wing flapping)
	Synchrony	Synchronised behaviour
	Light	Light schedule, intensity, quality
	Injuries	Trauma, pen fittings, culling, mutilations, predators
	Health status	Health status, esp. mortality & other disease prevalence (excl. lameness & skin problems)
	Guarantees	Welfare regulations (incl. e.g. enforcement) & owner qualifications (e.g. knowledge/skill/motivation/means regarding welfare)
	Health care	Health care measures like health plan, records, hygiene (contact to manure), bio-security & bird check frequency, farmer & vet qualifications regarding broiler health
	Other/new parameter(s)	Namely…

*: Preferred for assessment; colour coding: see S1 File.

#### Parameter level scoring, description and bar charts

Experts were asked to give a PLS (scale 0–10) for every combination of selected parameter and HS. Thus, the core output of the survey was a cross-table (Table 3, which was Table D in the survey, see S1 File) with a limited set of conventional and alternative HSs, their GWS, their breakdown in welfare-relevant parameters, WF and contribution to GWS expressed as PLS in the cells of the cross-table.

**Table 3:** (Table D in S1 File). Format of cross-table of housing systems, HSs, and given welfare scores, GWS, as column headings in green, taken from Table A in S1 File, and main welfare parameters, row headings in yellow with their weighting factors, WF, taken from Table F in S1 File. The task here was for the respondent to assign parameter level scores, PLS, on a scale from 0–10 (light blue field) for every parameter-HS combination. Inserted GWS and WF values represent average expert scores, showing only parameters that had n> = 2 PLS values for all HSs shown (see Table K (Summary table) in S2 File ‘Data exploration’).

Parameter\ HS Label	WF (0–10)	Battery cage*	Conventional US*	Conventional EU*	GAP 2	Organic US*	Freedom Food	Organic EU*	Better Life	Free range EU	Label Rouge II	Nature	Flock*	Hs Best 10	Hs Worst 0
GWS->		1.29	2.92	3.66	4.91	5.81	6.15	7.00	7.22	7.23	7.38	7.69	8.84	10	0
Density*	8.5	1.15	2.67	3.00	5.80	4.67	6.00	7.79	7.00	7.30	6.86	9.86	9.20	10	0
Breed*	8.3	2.09	3.00	2.39	3.00	4.25	6.00	7.12	6.00	6.57	7.67	9.86	8.25	10	0
Air quality	8.3	3.40	4.67	4.70	6.50	6.75	6.60	7.17	7.00	7.60	8.50	10.00	8.63	10	0
Litter*	8.2	0.18	3.57	4.06	5.00	3.75	6.83	5.85	8.13	7.00	6.20	9.50	8.14	10	0
Enrichment	6.9	0.33	1.50	1.09	5.25	6.75	6.00	7.42	7.00	7.95	6.58	10.00	8.96	10	0
Outdoor*	6.6	0.09	0.00	0.06	0.67	5.75	2.00	7.75	6.10	8.56	7.10	10.00	9.20	10	0
Space/pen*	5.6	1.45	2.92	3.33	6.00	6.00	5.50	7.55	7.50	6.50	5.60	9.80	8.67	10	0

*: Preferred for assessment; HsBest10 and HsWorst0 are logically constructed HSs that by definition have PLS of 10 and 0 respectively for all parameters; colour coding: see S1 File.

**Fig 1:**
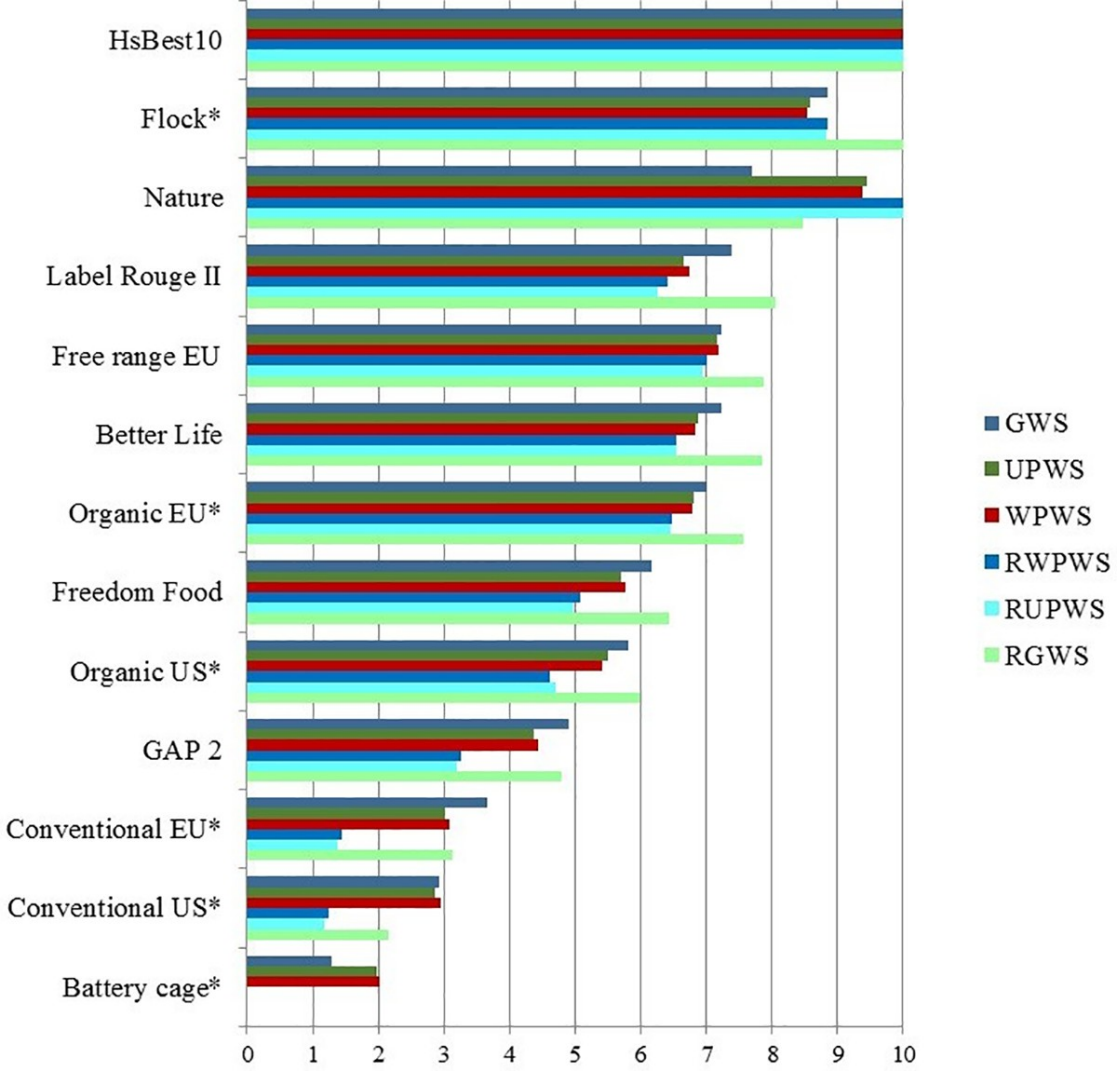
Format of bar chart showing the respondent’s welfare scores for selected housing systems (HSs). Example here based on average expert scores and average parameter level scores (PLS) presented as Table K in S2 File. Legend: GWS: Given Welfare Scores (in the survey showing the GWS of the expert-respondent; here showing average expert GWS values). UPWS: Unweighted Parameter Welfare Score (all WF = 1; sum of PLS/number of PLS) WPWS: Weighted Parameter Welfare Score (sum of (WF*PLS)/sum of WF) RWPWS: Rescaled WPWS (using the full scale 0–10 for the selected HSs) RUPWS: Rescaled UPWS RGWS: Rescaled GWS HsBest10: Logically best-possible HS; *: Preferred for assessment.

**Fig 2:**
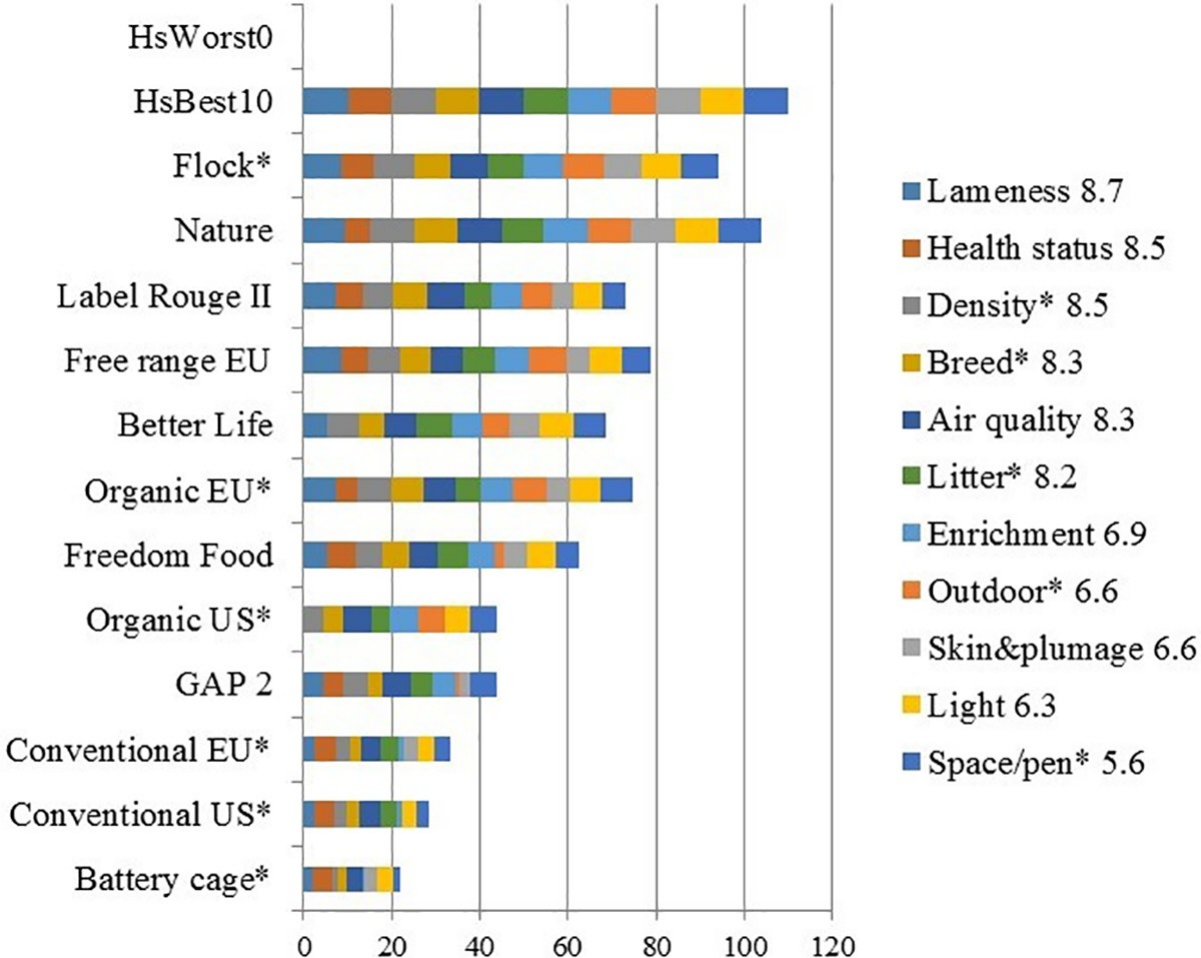
Format of bar chart showing the stacking of the respondent’s unweighted parameter level scores (PLS) for selected housing systems (HSs). Example based on average expert scores taken from Table K in S2 File.

**Fig 3:**
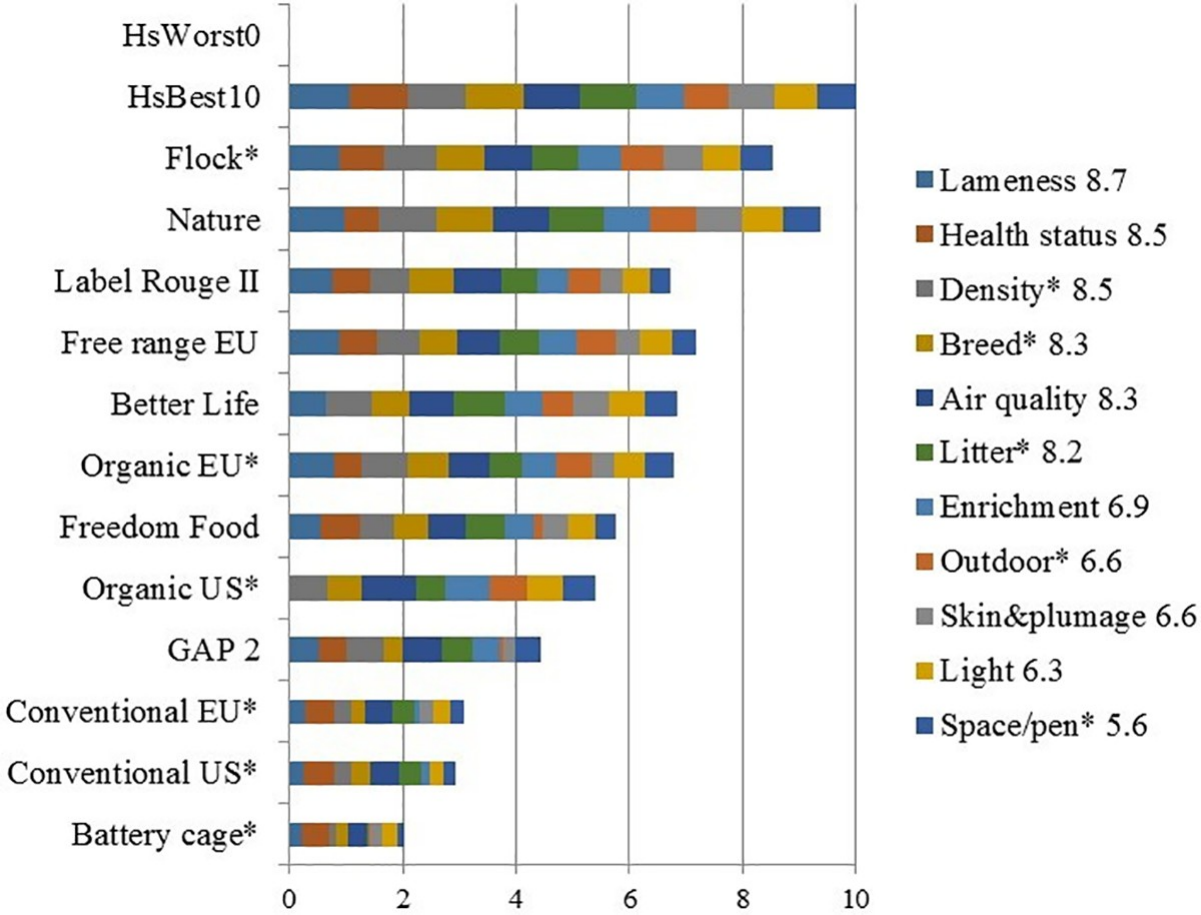
Format of bar chart showing the stacking of the respondent’s weighted parameter level scores (PLS) of selected housing systems (HSs). Example based on average expert WF scores taken from Table K in S2 File.

Note: UPWS, WPWS, RWPWS and RUPWS are all calculated welfare scores (CalcWS). ‘Rescaling’ (R) implies use of the whole scale, where the selected HSs with the highest and lowest scores were rescaled to 10 and 0 respectively, and the scores for the other HSs were transformed proportionally. As a consequence of rescaling the HS with the lowest score (here ‘Battery cage*’ is no longer visible, score 0).

The survey also contained a supplementary table (replicating Table D in S1 File; not shown here) where the respondent was requested to explain any major differences in PLS by inserting descriptive text, including a specification of what would correspond to a PLS of ~10 and ~0 if no HS had been assigned such a score. When scoring was complete, the expert’s attention was drawn to 3 bar charts (Figs 1–3 below). These showed how his/her GWS matched CalcWS derived from PLS with and without WF. In case of a (perceived) mismatch, the expert was allowed to modify GWS, WF and/or PLS values.

‘Unweighted’ implies that all WF were set at 1. The numbers shown in the parameter legend are the assigned WF (here: the average of the 23 experts that also provided valid PLS values). HsWorst0 and HsBest10 are logically worst and best-possible HSs respectively; *: Preferred for assessment. Since HsWorst0 has PLS = 0 for all parameters, it does not generate a bar in this chart. HsBest10 has PLS = 10 for all parameters, so it shows the maximum score that is logically, but not necessarily also under commercial conditions, possible. Since 11 parameters are included in this chart, the y-axis runs from 0 to 110 (= 11*10).

The numbers shown in the parameter legend are WF (here: the average of 23 respondents). HsWorst0 and HsBest10 are logically the worst and best-possible HSs respectively; *: Preferred for assessment. This chart shows that parameters with a high WF (such as Lameness, WF = 8.7) have a larger maximum bar (first blue bar on the left) compared to lower-weighted parameters (such as Space/pen*, WF = 5.6; darker blue bar on the right). Due to rescaling the y-axis runs from 0 to 10.

Finally, the experts were requested to state their area of expertise, especially regarding broiler welfare, and to identify up to 5 most knowledgeable experts for this kind of integrated broiler-welfare assessment, and to recommend up to 3 colleagues or other experts who might be willing to participate in the survey.

### Statistics

The available data posed a challenge for statistical analysis because it was not possible to collect hundreds of responses. The number of broiler welfare experts in the world is limited, the experts were only able to score partially overlapping subsets of HSs and parameters, and items did not have equal variance on the scales used (0–10). Therefore, we carried out extensive data exploration (see S2 File ‘Data exploration’), as well as simple pairwise t-test and sign test comparisons, because the primary objective of this study was to (semi-) quantify the welfare impact of adoption of (aspects of) alternative systems. Data exploration resulted in the decision to remove one frequently scored parameter (Space/pen*, n = 15; relatively low WF, high variance; repeated aberrant scoring as indicated by a lower PLS for Conventional US* than for Battery cage*) as well as the PLS scores of 2 out of 25 respondents (who had provided PLS scores) because their scores did not make sense logically and they showed a much lower and negative correlation between GWS and calculated scores (for further details see S2 File).

Unfortunately, expert concordance (Cronbach’s Alpha) could not be calculated in a formally correct way, because the dataset had too many missing values [31]. Provisional calculations and average Pearson’s correlation coefficients are reported in S3 File ‘Additional statistical analyses’.

#### REML

A REML (Linear Mixed Model) analysis was used to examine differences in GWS between HSs. For this analysis the complete dataset of GWS was used for every HS that was scored at least twice (S1 Dataset). Using the complete dataset implied that all GWS scores were included even when no explaining PLS scores were provided. The criterion of > = 2 GWS per HS implied that the minimum number was 6 for Modern cage and Concepts NL, and the maximum was n = 25 for Conventional EU*, out of the total number of 27 experts being involved in scoring (of which 2 did not provide PLS; see also S2 File).

In a preliminary REML analysis, the expert-related factors of Role (Scientist/Vet), Gender (F/M), Region (EU/nonEU) and HS were analysed, followed by a final analysis including HS alone.

A similar REML analysis was used to examine differences in WF between parameters, again using the complete set of WF values and for every parameter that was assigned > = 2 WF values. Here, again, 27 experts were involved in total and the number of WF per parameter ranged from n = 3 (for Preen/ComfB) to n = 25 (for Density* and Breed*). Here too, the effects of Role, Gender and Region were examined, as well as the effect of parameter in a separate analysis. Due to the limited dataset, interactions between expert-factors (Role, Gender, Region) and HS or parameter could not be analysed.

As an auxiliary measure of parameter importance we also calculated the Pearson correlation coefficient between the number of experts who selected a parameter by scoring it, and the predicted mean WF in the REML analysis using the complete dataset (of n = 27 experts), and the average WF values for the restricted dataset (n = 23 experts, using only WF values of parameters that had also been used to explain GWS by assigning PLS values).

Significant differences between HSs and parameters reported from the REML analyses are the result of posthoc pairwise comparisons using a Bonferroni correction.

#### ALM

An automatic linear modelling (ALM) analysis [32–34] was performed on the PLS values for each parameter (with > = 2 PLS values per parameter) and expert (n = 23 experts in total, excluding 2 experts that did not provide PLS, and 2 ‘outlier’ experts) to see to what extent parameters could explain the variance in GWS values (S1 Dataset). The criterion of at least 2 PLS values per parameter was used as this was the minimum number required for ALM, and because our objective was to use the complete dataset. However, this does imply that interpretation of the results requires taking into account the number of experts involved in scoring each parameter. In particular, when relatively few experts have scored a parameter significantly affecting GWS, caution is needed when recommending using this parameter to improve welfare. In ALM the variables (parameters) to be analysed are automatically prepared by searching for relationships within and between–independent–variables, grouping of variables, trimming outliers, transforming variables (for normalisation) and recoding procedures. The default ALM procedure in SPSS [34] was used, and this involved automatic data preparation and forward stepwise model selection, leading to a selection of variables and their predicted contribution to welfare (GWS).

We examined the contribution of WF in the ALM analysis, as including only PLS would imply explaining GWS from unweighted PLS values only, while using WF would imply explaining GWS from weighted PLS. This was done by calculating PLS fractions from PLS values and WF scores. Since the analyses using weighted PLS had little added value and were more difficult to understand, these are provided as background in S3 File. The main ALM presented is based on PLS. In order to further support this decision, we also carried out a trial ALM analysis using PLS values as well as WF values to see if the procedure would select WF as a factor (also see S3 File).

Since an important aim of this study was to determine how to best improve broiler welfare, we also classified all parameters as either input or output parameters for welfare (see Table 4). Most parameters were labelled as inputs, as these are inherently more suited to describe HSs, and because the inputs included all parameters that had a straightforward relation to an input, even when they were co-determined by animal presence or behaviour. As a result, parameters like Air quality, Heat, Cold, Foraging, Dustbath, Fd competition, Disturbance and Health care were all classified as input parameters. This left 5 output parameters, namely Lameness, Health status, Skin&plumage, Injuries and Preen/comfB. However, as the number of experts scoring the output parameters Injuries and Preen/ComfB was very low (n = 1 and n = 0 respectively), only 3 output parameters were left for the AML analyses (which required n> = 2 expert PLS assignments).

**Table 4:** Overview of parameters indicating which are output or input. The table shows arithmetic average WF scores, Standard Deviation, StDev, Minimum and Maximum values for only those parameters that have been used (by n = 23 experts) to explain given welfare scores (GWS) of housing systems (HS) using parameter level scores (PLS).

In/Output	Parameter label	Avg WF	StDev	Count	Min	Max
Output	Lameness	8.67	1.37	12	6	10
Output	Health status	8.55	1.29	11	6	10
Input	Guarantees	8.50	0.71	2	8	9
Input	Density*	8.48	1.69	21	4	10
Input	Water	8.33	2.08	3	6	10
Input	Breed*	8.28	2.11	18	2	10
Input	Air quality	8.27	0.90	11	7	10
Input	Litter*	8.19	1.07	18	6	10
Input	Health care	7.33	2.89	3	4	9
Input	Perch/rest	7.00	2.65	3	4	9
Input	Foraging	7.00	1.41	4	6	9
Input	Enrichment	6.88	1.58	17	4	9
Input	Outdoor*	6.61	2.56	18	2	10
Output	Skin&plumage	6.60	2.98	6	3	10
Input	Light	6.25	1.49	8	4	9
Input	Fd competition	4.50	2.12	2	3	6
Input	Group size	4.00	2.83	2	2	6
Input	Mother/family	4.00	3.61	3	1	8
Input	Synchrony	1.75	0.35	2	1.5	2

*: Preferred for assessment; The number of PLS per parameter is shown in column ‘Count’. Green colour coding is used to identify parameters that have been scored at least twice (required for the ALM analyses). Parameters that were scored more than 4 times (count > = 5) have a darker green colour.

A number of ALM analyses were performed in a specific sequence to unravel the probable contribution of parameters to GWS. We first used an ALM analysis to confirm that WF could be left out (reported in S3 File). We then used an analysis including HS as a factor to see which parameters generally affected welfare given the fact that HSs were clustered by the ALM procedure into welfare levels. Next, the HS factor was excluded to see which parameters best explained welfare. This is a primary output as the experts were requested to explain GWS as much as possible using all parameters selected for PLS scoring, so the main result should be an analysis that does not correct for the GWS level of the HS cluster. Subsequently, we repeated the latter two analyses (with and without HS) for the two subsets of input and output parameters separately to determine which were the main input and output parameters respectively, as they differ in the degree to which they are under human control to improve the birds’ welfare. Finally, an analysis was performed for each HS separately to determine which input or output parameters most affected the GWS of that particular HS, without taking into account the (PLS and GWS) scores given to the other HSs. Since this ALM analysis explains remaining variation in GWS from remaining variation in PLS within HSs across experts, it is only indicative of how to improve welfare within systems (and given the variation in GWS among experts). The results of the ALM analyses per HS and the analyses using either only input parameters or only output parameters (as well as the analyses using weighted PLS factions rather than unweighted PLS values) are reported in S3 File. The overall results have been summarised in the section ‘Summary tables’ of S3 File (Tables F and G in S3 File).

For each ALM analysis we report the explained variance in GWS values of the statistical model. The information criterion (AICc: Akaike information criterion for small sample sizes) is used to compare models. A lower information criterion indicates a better fit. We also report the types of variables, esp. clusters of HSs, (input/output) parameters and parameter-level clusters significantly explaining the variance in GWS as well as the contribution of each parameter (beta-coefficient). For example, a beta-coefficient of 0.5 implies that improving the PLS of that parameter by one unit (e.g. from PLS = 0 to PLS = 1) increases GWS by 0.5. When in the current dataset that parameter has a range of values from 0 to 10 (which may be the case, but not necessarily, see Table K in S2 File), then a beta-coefficient of 0.5 means that increasing PLS from 0 to 10 implies an increase in GWS score of 5 (= 0.5*10) welfare points. Note that in case of clustering a reference cluster is specified, and in that case the beta-coefficient indicates the number of GWS points compared to the reference (in the tables below and in S3 File). Finally, we report the ALM ‘importance score’, which indicates the relative importance of a parameter (between 0 and 1). Importance scores are computed by taking the utility range for each parameter separately and dividing by the sum of the utility ranges for all parameters [34]. The values thus represent proportions that add up to 1, indicating how much each parameter contributes to explained variance. For the ALM per HS accuracy % values are reported, indicating how accurately the statistical model for each HS predicts GWS.

## Results of the statistical analyses

### Factors affecting welfare scores and weighting factors using REML analyses

The REML analysis of experts’ Role, Gender and Region on GWS were not significant (Role: F_1,136.6_ = 0.26, p = 0.61; Gender: F_1, 147.2_ = 1.31, p = 0.26; Region: F_1,128.6_ = 0.20, p = 0.65).

**Fig 4:**
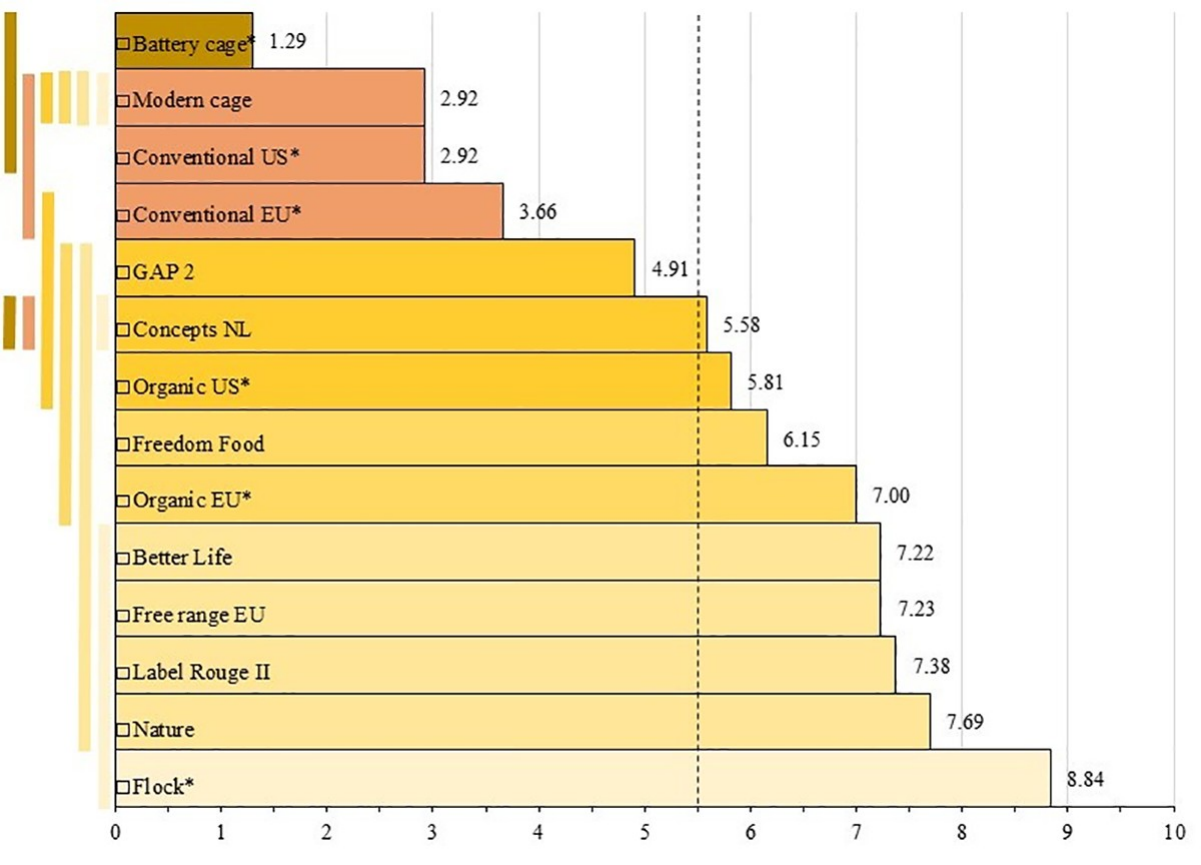
Significant differences between means of given welfare scores (GWS) of housing systems (HSs) based on the REML analysis. HSs with differently coloured vertical bars differ significantly (p< = 0.05). The dotted line indicates the cut-off for acceptability (5.5, in accordance with common usage on this scale, see the discussion); *: Preferred for assessment.

The REML analysis of HS effects on GWS was highly significant (F_13, 13.6_ = 70.1, p<0.001). Fig 4 shows the results of posthoc pairwise comparisons using a Bonferroni correction (more details in Table Aa in S3 File).

Fig 4 shows broadly 6 clusters of HSs differing in GWS level. The two HSs with the fewest number of scores (n = 6), namely Concepts NL (GWS: 5.6) and Modern cage (2.9), did not differ significantly from any other HS. Ignoring these two systems, we found that Flock* in Cluster 1 has a significantly higher GWS (8.8) than all HSs in Clusters 3–6. Cluster 2 (Nature, Label Rouge II, Free range EU, Better Life, GWS range: 7.7–7.2) did not differ from Clusters 3 and 4, but it did from Clusters 5 and 6. Cluster 3 (Organic EU*, 7.0; Freedom Food, 6.2) did not differ from 4, but it did from 5 and 6. Cluster 4 (Organic US*, 5.8; GAP 2, 4.9) did not differ from 5, except for Organic US* in Cluster 4 scoring significantly higher than Conventional US* (5.8 versus 2.9) in Cluster 5 (p = 0.027), but Cluster 4 did differ from Cluster 6. Cluster 5 (Conventional EU* and US*, 3.7 and 2.9 respectively) did not differ from 6 (Battery cage*, 1.3), except that Conventional EU* in Cluster 5 differed from Battery cage* in Cluster 6 (3.7 versus 1.3; p = 0.000).

The REML analysis of expert factors on WF values showed no effect of Role (F_1,131.7_ = 2.36, p = 0.13) or Region (F_1,145.3_ = 0.47, p = 0.49). Gender, however, was highly significant (F_1, 202.0_ = 15.8, p<0.001). On average, women assigned higher WF than men (predicted means of 7.2 and 6.4 respectively).

The REML analysis of (only) parameter effects on WF scores was significant (F_32, 10.3_ = 5.4, p = 0.003). Significant differences between pairs of parameters with and without Bonferroni correction are tabulated in Table Aa,b in S3 File. Only 5 significant differences remain after Bonferroni correction: Lameness (mean WF: 8.8), Heat (8.7), Health status (8.6), Injuries (8.5) and Litter* (8.3) each had a significantly higher WF than Dustbath (5.6).

As a supplementary measure of parameter importance, Pearson’s correlation coefficient for REML results between the number of experts that had provided WF and the predicted mean WF was low and not significant (R = 0.24, p = 0.17, n = 34). The Pearson’s correlation between average WF and number of experts (with n> = 2 experts per parameter and n = 23 experts in total) that had also used the selected parameters to explain GWS by providing PLS was modest and significant (R = 0.45, p = 0.04, n = 20).

### Regression analyses explaining overall welfare from component scores

#### All parameters and HSs

The ALM model using HSs as well as all (input and output) parameters as variables in the model explained 85.3% of the GWS variance (Information criterion 8.53). Significant differences between clusters of HSs explained most of the variation in GWS (Importance: 0.46) with smaller, but significant contributions from the parameters Health status, Density*, Health care, Enrichment and Breed* (Importance decreasing from 0.19 to 0.03; see Table 5). Experts mentioned the HSs in Cluster 5 (Conventional EU*, Conventional US* and Modern Cage) the most, and the ALM model used this as a reference (GWS Beta-coefficient 0). Cluster 6 containing Battery cage* scored a lower GWS (-1.6 welfare points compared to the reference Cluster 5), etc., while Flock* in Cluster 1 scored highest (+2.5). The most important contributing parameter was Health status. It had a beta-coefficient of 0.30, implying that increasing its PLS from 0 to 10 could potentially and generally add 3.0 points to the GWS (on a scale from 0 to 10). Note that the phrase ‘potentially and generally’ here means that this is true if this range of PLS (from 0 to 10) had *de facto* been used by the experts across the set of HSs. To verify this, the reader is referred to Table K in S2 File, where it may be noted that the average PLS values for Health status ranged from 4.4 for Battery cage* to 7.5 for Flock*, which thus covered only a third of the whole scale.

**Table 5:** Results of automatic linear modelling (ALM) regression analysis explaining given welfare scores (GWS) based on clustering of housing systems (HS) in welfare levels and parameter level scores (PLS) for all parameters (PLS with n> = 2 per parameter; total n = 23 experts).

Type of variable	(Transformed) variable	Beta	T	Signif.	Importance
Intercept		-1.606	-2.445	0.016	
HS cluster 1	Flock*	2.468	5.310	0.000	0.461
HS cluster 2	Nature, Label Rouge II, Free range EU, Better Life	2.048	5.474	0.000	0.461
HS cluster 3	Organic EU*, Freedom food	1.375	3.808	0.000	0.461
HS cluster 4	Organic US*, Concepts NL, GAP 2	0.936	2.625	0.010	0.461
HS cluster 5 (ref)	Conventional EU*, Conventional US*, Modern Cage	0.000	(Reference)		0.461
HS cluster 6	Battery cage*	-1.565	-4.822	0.000	0.461
Output parameter	Health status	0.299	5.018	0.000	0.186
Input parameter	Density*	0.236	4.410	0.000	0.144
Input parameter	Health care	0.347	3.870	0.000	0.111
Input parameter	Enrichment	0.143	2.985	0.003	0.066
Input parameter	Breed*	0.091	2.093	0.038	0.032

*: Preferred for assessment; Beta: Beta-coefficient; T: T-test result; Signif: Significance level (p value, see section ‘Statistics’, also for ‘Importance’); Ref: HS cluster reference (see text); darker green cells indicate higher importance values.

#### All parameters without HSs

The ALM model using all input and output parameters without HSs was the primary model used to determine which parameters affected welfare and by how much. This model explained 80.9% of the GWS variance (Information criterion 47.2). Most of the variation in GWS was explained by the significant parameters Density*, Health status, Enrichment, Health care and Litter*. Light was almost significant (p = 0.051). Their importance ranged from 0.41 to 0.03. Their maximum possible impact on GWS varied between 4.2 and 1.4 welfare points (see Table 6).

**Table 6:** Results of automatic linear modelling (ALM) regression analysis explaining given welfare scores (GWS) based on all parameter level scores (PLS with n> = 2 per parameter; total n = 23 experts).

Type of variable	(Transformed) variable	Beta	T	Signif.	Importance
Intercept		-4.23	-5.142	0.000	
Input parameter	Density*	0.421	7.905	0.000	0.409
Output parameter	Health status	0.383	5.897	0.000	0.227
Input parameter	Enrichment	0.266	5.656	0.000	0.209
Input parameter	Health care	0.397	3.782	0.000	0.094
Input parameter	Litter*	0.136	2.344	0.021	0.036
Input parameter	Light	0.141	1.965	0.051	0.025

*: Preferred for assessment; Beta: Beta-coefficient; T: T-test result; Signif: Significance level (p value, see section ‘Statistics’, also for ‘Importance’); darker green cells indicate higher importance values.

#### Input parameters without HSs

The ALM model using only input parameters explained 77.3% of the GWS variance (Information criterion 71.4). Most of the variation in GWS was explained by the significant parameters Density, Enrichment, Health care, Breed* and Litter* (see Table 7).

**Table 7:** Results of automatic linear modelling (ALM) regression analysis explaining given welfare scores (GWS) based on only parameter level scores (PLS) of input parameters.

Type of variable	(Transformed) variable	Beta	T	Signif.	Importance
Intercept		-2.92	-3.354	0.001	1.000
Input parameter	Density	0.371	5.886	0.000	0.383
Input parameter	Enrichment	0.252	5.040	0.000	0.281
Input parameter	Health care	0.459	4.027	0.000	0.179
Input parameter	Breed*	0.141	2.596	0.010	0.074
Input parameter	Litter*	0.134	2.013	0.046	0.045
Input parameter	Air quality	0.144	1.856	0.066	0.038

*: Preferred for assessment; Beta: Beta-coefficient; T: T-test result; Signif.: Significance level (p value, see section ‘Statistics’, also for ‘Importance’); darker green cells indicate higher importance values.

#### Output parameters without HSs

The ALM model using only output parameters explained only 39.4% of the GWS variance (Information criterion 204.4). Most of the variation in GWS was explained by the significant parameters Lameness and Health status (see Table 8). An increase in PLS of 1 unit (on the scale from 0 to 10) for Lameness and Health status added 0.64 and 0.54 welfare points to GWS respectively.

**Table 8:** Results of automatic linear modelling (ALM) regression analysis explaining given welfare scores (GWS) based on parameter level scores (PLS) of only output parameters and without clustering of HSs.

Type of variable	(Transformed) variable	Beta	T	Signif.	Importance
Intercept		-1.006	-1.342	0.182	
Output parameter	Lameness	0.638	7.540	0.000	0.697
Output parameter	Health status	0.547	4.973	0.000	0.303

*: Preferred for assessment; Beta: Beta-coefficient; T: T-test result; Signif.: Significance level (p value, see section ‘Statistics’, also for ‘Importance’); darker green cells indicate higher importance values.

## Discussion

### Main results

The objective of this study was to provide guidance for optimized improvement of broiler welfare based on expert-opinion elicitation and analysis. To this end we conducted an innovative, semantic-modelling type expert survey [4, 11, 35, 36]. Its purpose was to assess overall welfare and unravel the contributions of welfare parameters, especially in relation to conventional and feasible alternative broiler HSs. The international expert survey (response rate 38%; n = 27 experts in total responded) resulted in overall welfare scores (GWS) and component scores (PLS and WF) for 14 different HSs serving as benchmarks, including a negative control (Battery cage*) and a positive control (Flock*). The other tentative positive control HS, Nature, though not significantly different from Flock*, ended up in the second out of 6 clusters of HSs indicating different levels of welfare as judged by the experts.

The results of the survey have been condensed into three summary tables. The first summary table was previously presented partly in Table 3 in the Materials and Methods section above (and more fully in Table K in S2 File). Together with the bar charts (Figs 1–3), it showed main relationships between average GWS and calculated welfare scores, CalcWS. Two further summary tables were presented in S3 File ‘Additional statistical analyses’. Table F in S3 File shows an overview of the various ALM analyses. Most variance in GWS was explained in the ALM model using all available parameters and HSs (85.3%). Least variance was explained when only output parameters were included (39.4%). Understandably, ALM analyses with HS clustering in welfare levels had a higher percentage of variance explained than similar models without clustering. The latter, however, are more relevant to answer the question ‘how to best explain GWS *as much as possible* from PLS?’ Overall, the most frequently ALM-selected parameters were (presented in the order of importance, separated by;): Health status (output); Density*, Health care, Enrichment (all input); Breed* (input); Litter*, Air quality, Outdoor (all 3 input) and Lameness (output). The third summary table (Table G in S3 File) showed the REML results of differences between HSs in GWS clusters, and (absence of) differences in WF between parameters, combined with main ALM results, explaining GWS variance from input and/or output parameters across and within HSs without HS clustering.

A first question that arises from these summary tables relates to the birds’ welfare in these HSs. What is the best representation of overall welfare? On the one hand, we have collected GWS values from expert respondents. REML analyses showed how systems differed in GWS level (Fig 4). The clustering was similar to the clustering encountered in the ALM analyses, which is not surprising as these are both regression analyses. On the other hand, we presented CalcWS derived from component PLS and WF values, which were provided with the intention of explaining the expert’s GWS. In fact, CalcWS could be regarded as primary values too, perhaps to be explained or verified by GWS, rather than vice versa. This is how GWS was used in previous studies, namely to validate the semantic models SOWEL [4] and RICHPIG [35, 36]. The correlations between the two types of welfare scores were also high (R = 0.77–0.91; see Table J and Figs A-C in S2 File ‘Data exploration’). This suggests GWS and CalcWS may be considered as more or less equivalent. Thus, we have two potential ways to determine how welfare may best be improved. On the one hand, the beta-coefficients of parameters explain GWS variance in the ALM analyses. On the other hand, we have CalcWS based on average PLS with or without taking WF into account.

WF did not differ significantly between parameters, except that Dustbath had a significantly lower WF (5.6) compared to Lameness (8.8), Health status (8.6), Injuries (8.5) and Litter* (8.3). The former is a behavioural indicator strongly related to litter quality (hence classified as an input parameter), which has been associated with positive welfare [37]. The latter are mostly output parameters, except for Litter*, which has been associated with production and welfare problems, especially foot pad dermatitis [38, 39]. The absence of significant differences in WF between parameters was in line with the fact that the experts in the survey were only weighting main parameters selected to explain GWS. Also weighted CalcWS, i.e. using WF, did not show a better correlation with GWS than unweighted CalcWS from PLS alone (Figs A-C and Table J in S2 File). This is in accordance with what we found earlier in semantic-modelling studies [4, 8].

No effects were found of Region (EU/NonEU) or Role (Scientist/Vet) on GWS or WF, and there was no effect of Gender on GWS, but women provided significantly higher WF values than men (7.2 vs 6.4, p<0.001). The effect is not easy to explain. Perhaps, women find welfare more important, but then we would expect lower GWS scores. Previously, we found that women provided substantially higher welfare sores for indestructible enrichment materials for pigs, as well as some effect of Region [40], and the first semantic-modelling expert-opinion study [4] suggested an effect of Role in that ethologists seemed to provide lower GWS for pregnant-sow HSs than stress-physiologists. Hence, the type of expert may affect scoring for welfare. More large-scale studies seem required to better understand this. In addition, the selection criterion of what constitutes an expert remains an ongoing point of discussion. Our survey involved 27 respondents, including 20 senior broiler-welfare scientists and 7 veterinarians with an academic background. To obtain the best-possible set of respondents we used a kind of peer-review approach by asking experts to identify top experts in the field of study, and then to contact these persons as well (see S2 File ‘Data exploration’ for more details).

The main parameters explaining variance in GWS by PLS in the ALM analyses without HS clustering, were the output parameters Health status and Lameness, and the input parameters Litter*, Density*, Air quality, Breed*, Health care, Enrichment and Outdoor. However, the main ALM model is the model explaining GWS from all (and only) parameters. This, namely, was the task of the experts, to explain GWS fully based on PLS (and WF), without ‘correction’ for level of welfare (as was done in the ALM analyses with HS clustering). This leaves Health status (with a beta-coefficient of 0.38), Litter* (0.14), Density* (0.42), Health care (0.40) and Enrichment (0.27). Light was almost significant (p = 0.051; Table 6). The beta-coefficients indicate how much GWS would improve from 1 unit improvement in PLS for each parameter, so Litter* has a maximum potential impact of only 1.4 welfare points, while improving Density* from PLS 0 to 10 would improve GWS by 4.2 points. The impact of Health care should be interpreted with caution, as it was based on PLS values provided by only 3 experts. Another point to keep in mind is that parameters differed in the degree to which they vary across HSs (Table K in S2 File), and in the degree to which they can be changed. In general, inputs, like Litter*, Density* and Enrichment, are more under human control than outputs like Health status. With this in mind, stakeholders and decision makers should be able to determine how to most effectively improve the overall welfare of broilers across HSs based on the ALM analyses of this expert-opinion survey. However, further work may be needed to address the question how the PLS scales for the main parameters are to be interpreted. The experts often did not specify their parameter scales though a broad inference from general HS properties may give a first indication.

ALM analyses within HSs showed that overall welfare of Conventional EU* was affected mainly by Breed* and by Health status, potentially contributing 2.2 and 3.2 welfare points to GWS respectively. For Conventional US* Air quality and Litter* were selected, potentially contributing 2.6 welfare points, and 2.9 points for a PLS value of 7 instead of 2, 3 or 6. Such a result would seem to be in accordance with, for example, the finding that in the South-eastern US broilers had much lower incidence of severe footpad dermatitis (<5%) than broilers in the EU (13–63%) [41]. However, it should also be noted that a difference between PLS = 7 and PLS = 6 for Litter* in this survey was probably almost meaningless, as experts were assessing welfare across a much wider range using only a limited number of parameters.

Organic US* was affected by Outdoor* (2.9 welfare points improvement from PLS = 0 to PLS = 1), which may make sense in that pasture access is not required for Organic US* production. Organic EU* was affected by Lameness and Health status. Density* was selected for both Battery cage* (low-end welfare), and Nature and Flock* (high-end welfare). Density* potentially impacts Nature considerably (4.3 points from PLS = 9 to 10). Here too, the difference was very small, and potentially meaningless. It is probably a consequence of the fact that the statistical ALM model per HS explains variance in GWS of each HS separately based on PLS values of that HS alone. So, it does not, for example, say what would be the welfare contribution of providing outdoor access to birds in Battery cages*, as that would have to be based on (the range of) PLS values assigned across HSs (discussed above).

The alternative route to determine how best to improve broiler welfare would be to focus on CalcWS and derive potential welfare impact from average (weighted or unweighted) PLS values as presented partly in Table 3 of the Materials and Methods, and more fully in Table K in S2 File. There, the main parameters are Lameness, Health status, Density*, Breed*, Litter* and Air quality (all with average WF >8). Other, somewhat less important parameters are Enrichment and Outdoor*. Space/pen* had a relatively low weighting (5.6) and also had PLS values that did not make sense logically (a higher score for Battery cage* than Conventional US*), leading to a disqualification of this parameter for the statistical analyses. Other important parameters were the output parameters Lameness and Health status (WF >8.5) and Skin/plumage (6.6), and the input parameter Light (6.3). However, these had missing values (n <2) for one or two of the 12 main HSs, from which Modern cage and Concepts NL had been excluded as these were scored the least (n = 6), and, probably as a result, were not significantly different from any other HS (Table A in S3 File). Average PLS values for these 11 main parameters more or less covered the whole scale (0–10), with the exception of Health status, which ranged from 4.4 to 7.5, and possibly also Air quality and Light (which ranged from 3.4–10 and from 3.1–9.8 respectively; see Table K in S2 File). This range is relevant to assess the potential impact using the beta-coefficients in the main ALM analysis, and when doing so, it is therefore strongly advised to always aditionally compare the expected benefits with a direct welfare calculation using the average PLS values (with or without weighting using average WF values).

### Survey subjectivity versus empirical studies

Our results are based on an expert survey, and despite the involvement of a fairly substantial number (n = 27) of international welfare experts, this survey may nevertheless be criticized for being subjective and based on perception, this being a frequently-heard objection. For example, a previous expert survey was criticised for not being ‘scientific’, before it was published [40]. In that paper we, too, fully acknowledge that empirical studies are important, especially when there are differing opinions and firmly-held beliefs. Experts participating in this broiler survey regularly (n = 16) also complained about it being difficult, some even called it ‘daunting’. Indeed, it was not an easy task, as we were not so much interested in the experts’ subjective opinions as we were in their rational and science-based welfare assessment. In semantic modelling this can be formalised and thus made explicit [4, 5, 8, 12, 35]. In the current, innovative, semantic-modelling type survey, we focussed on the task of explaining overall scores (GWS) based on component scores (PLS and WF), and we provided the experts with an Excel tool that graphically showed how GWS related to PLS and WF as a ‘double check’ (Figs 1–3). The focus on science-based welfare assessment was also reflected in our decision to discard the parameter Space/pen* and two ‘outlier’ experts (which had negative correlations between GWS and CalcWS (see S2 File). In the end, logic was used as a decisive criterion whether or not to disqualify scores as we felt we had to refrain from making recommendations for welfare improvement that were based on data that was known to be unreliable.

Experts appeared to ‘behave similarly’ (see especially Fig A in S2 File), even though a tentative Cronbach’s Alpha provided a value of only 0.58 for GWS and 0.79 for WF (1.0 being optimal). This differs from previous studies where GWS for HS generally had a much higher Cronbach’s Alpha value than WF [4, 36]. We think these relatively low Cronbach’s Alpha values could be due to the missing values in the current datasets (which were all substituted by average values to be able to calculate Cronbach’s Alpha). The average Pearson’s correlation coefficient between all pairs of scores without substitution of missing values (0.80 for GWS and 0.28 for WF) appeared to be a more reliable, though still provisional, indicator of expert concordance in the current study. These values are also more in accordance with what we found in earlier studies [4] (see also Figs A and B in S2 File). The relatively low correlation for WF may have been aggravated in this survey by the fact that we only asked to weight the 4 or 5 main parameters (which would then all be expected to be ‘most important’). This may have resulted in some variability between experts in scaling (for instance by experts more or less zooming in on relatively small differences between selected parameters).

Two alternatives may be proposed for this kind of survey regarding welfare assessment. Alternative 1) would be to construct a semantic model, which to date is not available for broilers. Since we didn’t have the resources to do both, it was decided to do the survey as it appeared more feasible, but a semantic model might well have been a valuable and realistic alternative. In fact, we constructed the outline of a preliminary model which we used as a basis for the current survey, and we agree with Collins et al. [42] that farm animal welfare could benefit from more modelling.http://www.mdpi.com/2076-2615/8/4/53 Alternative 2) would be to carry out empirical research. In the case of overall welfare assessment this may require visiting many (randomly selected) farms (many times), taking many animal-based welfare measures and then integrating the results into an overall welfare assessment. This certainly is an even much more ‘daunting’ and costly task than conducting a semantic-modelling type expert survey. This approach was attempted in the Welfare Quality (WQ) project [22, 23, 29, 43–46]. However, the validity of the WQ premise that welfare assessment should be based as much as possible on only animal-based measures has been challenged [27]. From a semantic-modelling perspective, all available knowledge should be used to achieve the best possible assessment, including scientific knowledge about how animal-based and resource-based, i.e. output and input parameters for welfare relate to each other. Experts can be presumed to be the most knowledgeable in this respect, and would, it is assumed, have applied this knowledge, consciously or unconsciously, in their welfare assessment as elicited in the current survey. In this study, we found that the percentage of variance in GWS scores that could be explained dropped from 81% when all parameters were used, to 39% when only output parameters were used (both without HS clustering). Using only input parameters resulted in 77% explained variance. This suggests that the experts were clearly using input parameters (design criteria, resource-based measures) as well as animal-based output measures to assess welfare. On average, each expert used 7.1 input parameters and 1.3 output parameters (n = 23), while the number of outputs per expert ranged from 0 (n = 6) to 3 (n = 3 experts), and no expert used only output parameters to explain GWS. The preference for input parameters may be related to the fact that HSs are described in input terms, and to the fact that a number of input parameters were identified as preferred for assessment (by *), even though experts were explicitly allowed to (exclusively) choose outputs as well. The survey thus seems to support the thesis from semantic modelling that for overall welfare assessment all available information should be used, including both input and output parameters, and their known relationships. That said, however, we do recognise the importance of output measures, and this was also reflected in the survey in that, though not selected most often and though not significantly different, the output parameters Lameness and Health status had a higher mean WF (8.8 and 8.6) than the other (more often) selected main input parameters (Table Aa in S3 File).

A remarkable finding based on WQ indicators was reported by Gocsik et al. [22], namely that the high-end market systems Organic EU* and Free range EU, labelled ‘Extensive outdoor’ in their publication, had a lower WQ index score (698 and 733 respectively) compared to middle-market systems (Volwaard and Puur & Eerlijk; WQ index score 736), which appear similar to Better Life and perhaps Concepts NL in the current survey. We did not find significant differences between these systems, and, while the mean GWS for Organic EU* (7.0^bc^), Better Life (7.2^ab^) and Free range EU (7.2^ab^) were rather similar, like the WQ index, the scores were a little lower in the case of organic. Concepts NL, which seems similar to Puur & Eerlijk, but with a higher density, no veranda or daylight as in Puur & Eerlijk, scored substantially lower (5.6^abcdef^), though this difference was not significant, probably related to a low number of scores (n = 6; Table A in S3 File).

A final set of concerns expressed regarding the WQ protocol for broilers is that it has a poor sensitivity, that it is rather conservative (since it reduces, for example, to the resources water access and stocking density in the absence of sensitive animal-based parameters), and that, perhaps most importantly, WQ assessment and scoring, when applied on real farms, categorises most intensive farms as ‘acceptable’ (88%, n = 42 flocks; 23 farms) [23]. In a way, this may appear to be in accordance with the fact that most consumers are buying ‘conventional’, thus suggesting general ‘acceptability’. However, in our survey, conventional HSs received mean welfare scores of 3.7^de^ and 2.9^ef^ for typical systems used in the EU and the US respectively. This is clearly ‘unacceptable’ on a 10 point scale where 5.5 is commonly used as the cut-off point of what is acceptable [35, 40]. It may be noted that the experts in our survey were assessing animal welfare, defined as what matters to animals from their point of view. This is not to be confused with what matters to us humans (e.g. consuming affordable meat), or what is morally acceptable, taking all interests into account. With this criterion (5.5 as a cut-off) for broiler welfare, also GAP 2 must be regarded as unacceptable (mean: 4.9^bcd^), while Concepts NL (5.6^abcdef^) and Organic US* (5.8^bcd^) may just be acceptable, though only barely, and given the individual variation many individual farms of these types of HS will fall below the minimum standard as indicated by the experts (Fig 4). So, these would be the systems that could benefit the most from efforts to improve welfare by improving the aspects identified in this paper. At the high end of our scale are the two positive control systems, the semi-natural backyard Flock* (8.8^a^) and Nature (7.7^ab^), as well as several existing systems (Label Rouge II, 7.4^ab^; Free range EU, 7.2^ab^, Better Life, 7.2^ab^ and to a lesser extent Organic EU*, 7.0^bc^). These may serve as benchmarks for improvement. Another notable point is that the Modern cage did not receive a score that would suggest it has much viability from a welfare-experts’ point of view (2.9). At present, cages are not allowed in the EU, and only a very small number of farms in the world use a cage system for broilers (though there are some developments [47, 48]), and this may have affected the experts’ judgement on this HS.

As regards the tentative positive-control system Nature, we found that it had a relatively high variance (REML standard error 0.57 despite n = 13). Three experts gave a GWS of less than 5.5 to Nature, thus apparently being *de facto* unacceptable to them. Naturalness is a somewhat vague and ambiguous concept [49], and living under natural conditions may be inherently conflicting for welfare assessment, in that it may be regarded as providing both high and low welfare at (almost) the same time. It is not clear whether animals in nature really have high welfare, even though this may appear to be the case when the normal focus is on welfare problems in intensive systems. This implies that more in depth (philosophical/rational) thinking about welfare, as well as making welfare assessment more explicit, as was done to a limited extent in this survey, and providing empirical evidence about welfare under natural conditions, may all play a constructive role in the welfare debate.

## Conclusions

This semantic-modelling type expert elicitation provides a science-based and (semi-) quantified decision-support tool to help stakeholders, such as chain actors, policy makers and NGOs, make value-for-money decisions regarding the improvement of broiler welfare. The contribution of welfare components to overall welfare has been quantified in two ways: a) using the beta-coefficients of statistical regression (ALM) analyses to explain variance in GWS based on variance in PLS, and b) using a semantic-modelling type (weighted average) calculation of overall scores (CalcWS) based on component (PLS and WF) scores. The two methods, GWS and CalcWS, appear to be equivalent, as their results are highly correlated, and it is recommended that both be used in concordance.

Typical conventional broiler housing-and-management systems in the US and the EU received rather inadequate overall welfare scores, and it is important to improve this, not just by trial and error, but in the most efficient way possible. The current study provides a basis for such an evaluation and improvement. Alternative systems already exist, and aspects to most effectively improve welfare across housing systems include both input parameters like Density, Litter and Enrichment, and the output parameter Health status.

## Supporting information

S1 File Survey, invitation and background. (DOCX)

S2 File Data exploration. (DOCX)

S3 File Additional statistical analyses. (DOCX)

S1 Dataset Datasets used for statistical analyses (REML and ALM in SPSS). (XLSX)
