# Supplementary material for: Broiler welfare trade-off: A semi-quantitative welfare assessment for optimised welfare improvement based on an expert survey
Source: PLoS One. 2019 Oct 1;14(10):e0222955. doi: 10.1371/journal.pone.0222955 (PMC6772121; doi:10.1371/journal.pone.0222955)
Supplement: S1 File — (DOCX) [file pone.0222955.s001.docx]

## S1 File. Survey, invitation and background

This file contains the following sections: Survey, Invitation, Pilot interviews and Listing housing systems and parameters. The latter sections provide some supporting information about the bio-logic underlying the construction of the survey.

### Survey

LS,

Please find the survey below. If you need help, my contact details are: [marc.bracke@wur.nl](mailto:marc.bracke@wur.nl) or phone: +31 317 480558.

You are requested to share your views on integrated welfare assessment of chickens kept for meat production at the housing and management ‘systems’-level. The survey involves providing overall welfare scores for 5-6 housing systems/farms, weighting factors and scores for 4-5 welfare parameters (all on a scale from 0 to 10), thus tentatively expressing what kind of welfare improvement may be expected from adopting specified alternative systems. Your answers will be kept strictly confidential.

1. First, select 5-6 housing systems (HSs) from Table A by assigning a given welfare score (GWS; decimals allowed). Try to cover the whole scale, ie include at least 1 low-welfare HS (GWS< 3) & 1 high-welfare HS (GWS>7). Tentative scores are fine & may be modified later. Scores reflect your current opinion, so they cannot be 'wrong'. Generally, focus on 'average farms', typical example/representative farms with an 'average' welfare status. Focus on systems you and other broiler-welfare experts are capable of assessing. Focus on (more or less) feasible alternative (enhanced-welfare) systems compared to conventional. Alternative HSs include organic farming & schemes defined by farmers (eg NCC), retailers, food chains and/or NGOs. In total preferably 5-6 HSs should be scored (1 conventional, 1-2 alternatives, 1 high, 1 low welfare HS).

2. You may also specify new HSs in Table A by supplying a new label (max 18 characters) for HS1 (etc) and replacing 'Namely...' with keywords characterising the HS.

##### S1 Table A (below). Select & score 5-6 housing systems (HS) for welfare (GWS).

| **GWS (0-10)** | **HS label** | **HS description (keywords)** *: preferred systems for assessment HS1-HS6 can be used to specify new systems (eg AB free; McDonalds, etc). |
| --- | --- | --- |
|  | Conventional US* | Average/typical conventional farm in the US* |
|  | Conventional EU* | Average/typical conventional farm in the EU* |
|  | Battery cage* | Average/typical (traditional) battery cage for broilers* |
|  | Modern cage | Modern colony cage (nylon wire, in-house hatching, automated harvesting) |
|  | Organic US* | Average/typical organic farm in the US* |
|  | Organic EU* | Average/typical organic farm in the EU* |
|  | Flock* | Small backyard flock with mother hen, semi-natural, quality housing & (health) care* |
|  | Nature | Wild jungle fowl living in a fully natural environment |
|  | Freedom Food | Freedom food/RSPCA assured (average farm) |
|  | Better Life | Better Life scheme of Dutch Society for Protection of Animals (average farm) |
|  | Concepts NL | NL retailer scheme(s) (eg AH; 'middle segment' between conventional & Better Life) |
|  | Free range EU | Average/typical free-range farm in the EU |
|  | Label Rouge II | Label Rouge type II (average farm) |
|  | GAP 2 | Global Animal Partnership step 2 |
|  | HS1 | Namely.... |
|  | HS2 | Namely.... |
|  | HS3 | Namely .... |
|  | HS4 | Namely .... |
|  | HS5 | Namely .... |
|  | HS6 | Namely .... |

Optional: Refine grey HSs of Table A (& Table C) in (the green cells of) Table B.

But first briefly check Table C & note you may also specify HSs in more detail when explaining parameter scores later. Table C is a guide and may contain errors. Plz correct (only) major errors using Tbl B but don't waste valuable time on this.

##### S1 Table B. To modify the description of predefined HSs (optional & first see Table C below)

| **HS label** | **Housing system (HS) refined description (keywords)** |
| --- | --- |
| Conventional US* |  |
| Conventional EU* |  |
| Battery cage* |  |
| Modern cage |  |
| Organic US* |  |
| Organic EU* |  |
| Flock* |  |
| Nature |  |
| Freedom Food |  |
| Better Life |  |
| Concepts NL |  |
| Free range EU |  |
| Label Rouge II |  |
| GAP 2 |  |

##### S1 Table C (below). More extended example descriptions of housing systems (HS); *: preferred HSs;~: about. Note: Indicative description only; you have to fill in/infer ‘missing values’ yourself (may modify using Table B).

| **HS label** | **HS description** |
| --- | --- |
| Conventional US* | Average/typical conventional farm in the US*; ~20.000ft/1860m2/house; 32-43g/m2; 6.5-9lbs/ft2; ~22k birds/house; fast-growing (Cobb): 58gr/d; ~ 2.7kg BW in ~46d; wood shavings;1x/day checked; litter reused in multiple rounds; natural/tunnel ventilation; |
|  | no enrichment (besides litter); no outdoor; dim light;controlled light, typically 23:1 L:D day1-7, >day7: 20:4 L:D; ~4.5% mortality; no welfare legislation. |
| Conventional EU* | Average/typical conventional farm in the EU*; ~22birds/m2;~33-42kg/m2 (6.8-8.5 lbs/ft2; ~10-20k birds/house; fast-growing: ~55-60.5gr/d; ~ 2.3kg in 35-40d; woodshavings-based litter on concrete; no enrichment (besides litter); no outdoor; severe pododermatitis: 20-30%; thermal control; thinning; 6h/d dark (4h continuous; EC Directive); fully indoors; ~3-5% mortality. |
| Battery cage* | Average/typical (traditional) battery cage for broilers*; ~45kg/m2; ~30birds/m2 @1.5kg; ~400cm2/bird; group of ~6 birds/cage; fast growing; metal wire floor & cage; no enrichment; no outdoor. |
| Modern cage | Modern colony cage (nylon wire floor above manure belt, in-house hatching, automated harvesting); ~18birds/m2; ~45kg/m2; up to 4 tiers; colony of ~100 birds/cage; ~25k birds/house; fast-growing: ~64g/d; ~2.7kg BW in~40d; no enrichment; no outdoor; thermal controlled building; 2-6% mortality. |
| Organic US* | Average/typical organic farm in the US*; not regulated; outdoor may be veranda/porch/ moveable coop; no specific welfare requirements (only organic food & treatment). |
| Organic EU* | Average organic farm in the EU*; <1600m2/enclosure; fixed housing (≤10 birds/m2 & 21kg/m2)+≥4 m2/bird outdoors; <4.8k birds per enclosure; slow-growing: ≥81d;2.6kg in 70d~37gr/d(NL); >1/3 solid floor with litter eg straw/wood shavings; enrichment grain or straw; ≥1/3of life outdoor; mainly covered by vegetation & cover & easy access to food & water outdoors; roughage or silage provided daily; severe FPD ~60%; natural ventilation; perches; ≥8 h continuous dark; mortality 3%; EU regulations enforced (~1x/yr unannounced visit); immediate, allopathic treatment, records kept. |
| Flock* | Small backyard flock, with mother hen & cock, extensive semi-natural environment, quality housing & (health) care*; stable family group, ~10 adult birds/10k m2 backyard with vegetation & cover; local breed; balanced diet; protected from predators. |
| Nature | Wild jungle fowl living in a fully natural environment. |
| Freedom Food | Freedom food/RSPCA assured (average farm); 30kg/m2; 19birds/m2; ≤10k birds/enclosure; No limit to strain or slaughter age; some enrichment (besides litter); no outdoor; 1 nipple/10birds, 1 cup/28birds; 25mm linear or 16mm circular feederspace/bird; >8h light;>20lux;≥6h continuous dark/d; 14h light >5d. |
| Better Life | Better Life scheme of Dutch Society for Protection of Animals (average farm); ~25-30kg/m2; 12birds/m2 +167cm2/bird covered veranda/porch (≥20% of total area;167cm2, 35d used); large group; slower growing: 45g/d; 2.3kg in ~56d; Concrete & woodshavings; enrichment: 2g grain spread (from d15) or 1 strawbale/1000birds; 100%plant-based, ≥70% grains; severe footpad dermatitis ~3-3%; 8h/d dark; ≥20 lux natural light; mortality: ~3%. |
| Concepts NL | Dutch retailer scheme(s) (eg AH; 'middle segment' between conventional & Better Life); -33-38kg/m2; 14-19birds/m2; large group; Slower growing: 49g/d; 2.4kg in ~46d; eg Hubbard JA 987; concrete, woodshavings; enrichment: 2g grain spread (as of d15); 1 strawbale/1000birds (or 1 pecking stone/200m); no outdoor; 6h/d dark; daylight; ~2.5% mortality. |
| Free range EU | Average/typical free-range farm in the EU; 19birds/m2 or 27.5kg/m2 indoor + 1 m2/bird outdoor (ie avg 0.95bird/m2); 2.1kg in 56d; enrichment grain or straw; 50% of life outdoor access; ≥70 gr grain in feed; Severe FPD ~3-5%; daylight; 8 h dark; mortality: ~2.5%. |
| Label Rouge II | Label Rouge type II (average farm); 20 birds/m2+ outdoor(2m2/bird) (ie avg 0.5 bird/m2 ~ 1kg/m2); ≤1k birds/enclosure; slow growing (eg Hubbard): >81d;selected for breeding & body composition (breasts, low fat, thin skin); no animal food, >75% cereals. |
| GAP 2 | Global Animal Partnership step 2; ~32kg/m2; large group; fast-growing: 58g/d; 2.7kg BW in 46d; woodshavings; 1 enrichment (eg straw bale)/70m2; no outdoor; 1% of total diet grain in feed; 8h/d dark; some natural light via ventilators. |

3. Next, copy all scored HS labels from Table A in the green cells of Table D, sorted more or less by GWS (low to high welfare).

##### S1 Table D. To assign parameter scores (0-10; light blue field) after selecting housing system labels (green) & GWS (darker blue) from Table A (above), and parameter labels (yellow) & weighting factors (WF, brown) from Table E (below).

| **P#** | **Parameter\HS Label** | WF (0-10) | HS1 | HS2 | HS3 | HS4 | HS5 | HS6 | HS7 | HS8 | HS9 | HS10 | Hs Best 10 | Hs Worst  0 |
| --- | --- | --- | --- | --- | --- | --- | --- | --- | --- | --- | --- | --- | --- | --- |
| Nr | GWS-> |  |  |  |  |  |  |  |  |  |  |  | 10 | 0 |
| 1 | 1^st^ main parameter |  |  |  |  |  |  |  |  |  |  |  | 10 | 0 |
| 2 | Etc |  |  |  |  |  |  |  |  |  |  |  | 10 | 0 |
| 3 |  |  |  |  |  |  |  |  |  |  |  |  | 10 | 0 |
| 4 |  |  |  |  |  |  |  |  |  |  |  |  | 10 | 0 |
| 5 |  |  |  |  |  |  |  |  |  |  |  |  | 10 | 0 |
| 6 |  |  |  |  |  |  |  |  |  |  |  |  | 10 | 0 |
| 7 |  |  |  |  |  |  |  |  |  |  |  |  | 10 | 0 |
| 8 |  |  |  |  |  |  |  |  |  |  |  |  | 10 | 0 |
| 9 |  |  |  |  |  |  |  |  |  |  |  |  | 10 | 0 |
| 10 |  |  |  |  |  |  |  |  |  |  |  |  | 10 | 0 |

4. To explain (the differences between) the GWS scores, select 4-5 main welfare parameters in Table E by assigning a weighting factor (WF) from 0 (not important) to 10 (most important) (decimals allowed).

Optional: Give some (lower) WF scores to several moderately and unimportant parameters.

Also try to provide WF scores for preselected parameters (marked by *)

5. You may also specify new parameters in the light-yellow cells in Table E.

Table 6 allows refining grey parameters in Table E and/or explain the WF scores (using the parameter label or P# in Table D where you are requested to insert the selected parameters & WF scores).

6. Insert the main parameters (labels) and WF scores in Table D.

7. Assign parameter scores (0-10) for each HS-parameter combination in the light-blue field of Table D.

##### S1 Table E. List of parameters (Para). *: preferred for assessment

| WF | **Para label** | **Parameter description (may alter yellow cells below)** |
| --- | --- | --- |
|  | Space/pen* | Space/pen (m2); total enclosure size (indoor & outdoor) |
|  | Density* | Stocking density (eg kg/m2) |
|  | Group size | Social contact (group size) |
|  | Breed* | Breed (esp growth rate) (and other type-of-bird-related characteristics) |
|  | Litter* | Floor quality (presence of litter; litter quality) |
|  | Air quality | Air quality (fresh air, dust, NH3, humidity) |
|  | Enrichment | Enrichment/stimulation, eg straw bales, strings, platforms etc |
|  | Outdoor* | Outdoor access & quality of outdoor area |
|  | Foraging | Ability to search for food, eg scratching for grains in litter |
|  | Water | Water quantity, quality, drinker type |
|  | Fd level (E) | Feeding energy level/schedule/system |
|  | Fd quality | Food quality nutritionally (other than energy) & food hygiene |
|  | Dust bath | (Quality of) dustbathing ability |
|  | Fd selection | Ability to select ingested food items, e.g. variation in food items & palatability |
|  | Lameness | Lameness and other locomotion problems |
|  | Skin&plumage | Skin problems & plumage condition |
|  | Heat | Exposure to heat stress & general thermal regulation |
|  | Cold | Exposure to cold stress |
|  | Fd competition | Competition for food |
|  | Group stability | Social stability (eg mixing) |
|  | Handling | Handling and other issue related to fear of humans |
|  | Disturbance | Disturbance eg predation/panic (non-human/social) |
|  | Cover | Cover to hide (eg for predators and conspecifics) |
|  | Moveability | Ability to move around & movement comfort |
|  | Mother/family | Mother hen presence, family group, group composition |
|  | Find fd&water | Ability to learn to find food en water at an early age |
|  | Perch/rest | Perching & resting comfort (presence, perch quality, length) |
|  | Preen/comfB | Preen/comfort behaviour (eg wing flapping) |
|  | Synchrony | Synchronised behaviour |
|  | Light | Light schedule, intensity, quality |
|  | Injuries | Trauma, pen fittings, culling, mutilations, predators |
|  | Health status | Health status, esp mortality & other disease prevalence (excl lameness & skin problems) |
|  | Guarantees | Welfare regulations (incl eg enforcement) & owner qualifications (eg knowledge/skill/motivation/means regarding welfare) |
|  | Health care | Health care measures like health plan, records, hygiene (contact to manure), bio-security & bird check frequency, farmer & vet qualifications regarding broiler health |
|  | Parameter A | Namely ... |
|  | Parameter B | Namely ... |
|  | Parameter C | Namely ... |
|  | Parameter D | Namely ... |
|  | Parameter E | Namely ... |
|  | Note: Parameters in yellow cells can be altered (free to specify whatever you need). | |
|  | Parameter labels should not be longer than 18 characters. | |
|  | Table F can be used to specify/redefine weighted parameters & explain weightings. | |

##### S1 Table F. Footnotes to add more text to characterise parameters (grey in Tbl E above) & explain weightings

| P# | **Parameter label** | **Parameter characterisation & explanation of weighting** |
| --- | --- | --- |
|  |  |  |
|  |  |  |
|  |  |  |
|  |  |  |
|  |  |  |
|  |  |  |
|  |  |  |
|  |  |  |
|  |  |  |
|  |  |  |
|  |  |  |
|  |  |  |
|  |  |  |
|  |  |  |
|  |  |  |

8. Explain (major differences in) parameter scores assigned in Table D by inserting text (keywords) in Tables Db-Dc & also indicate what would be ~10 or ~0 scores if no HS has been assigned either a high or low parameter score (in the columns ‘ HsBest10 and HsWorst0 respectively). Note: HsBest10 and HsWorst0 by definition receive parameter scores of 10 and 0 for each weighted parameter. Though often not possible in reality, these systems are a reminder to explain the whole parameter scale (0-10). While assigning parameter scores in Tbl D, you are allowed to revise selections from Tbl A (HS, GWS) and Tbl E (Parameters, WF).

[for your convenience and before proceeding to the final part of completing Table D, you may now first want to copy Table D here; this is because Tables 4b & 4c below can be seen as (right-hand) parts of one larger ‘Table D’.]

##### S1 Table Db

| **P#** | **Parameter Label** | HS1 | HS2 | HS3 | HS4 | HS5 | HS6 |
| --- | --- | --- | --- | --- | --- | --- | --- |
| 1 |  | Here specify what is true for this parameter & HS (etc) |  |  |  |  |  |
| 2 |  |  |  |  |  |  |  |
| 3 |  |  |  |  |  |  |  |
| 4 |  |  |  |  |  |  |  |
| 5 |  |  |  |  |  |  |  |
| 6 |  |  |  |  |  |  |  |
| 7 |  |  |  |  |  |  |  |
| 8 |  |  |  |  |  |  |  |
| 9 |  |  |  |  |  |  |  |
| 10 |  |  |  |  |  |  |  |

##### S1 Table Dc

| **P#** | **Parameter Label** | HS7 | HS8 | HS9 | HS10 | HsBest10 | HsWorst0 |
| --- | --- | --- | --- | --- | --- | --- | --- |
| 1 |  |  |  |  |  |  |  |
| 2 |  |  |  |  |  |  |  |
| 3 |  |  |  |  |  |  |  |
| 4 |  |  |  |  |  |  |  |
| 5 |  |  |  |  |  |  |  |
| 6 |  |  |  |  |  |  |  |
| 7 |  |  |  |  |  |  |  |
| 8 |  |  |  |  |  |  |  |
| 9 |  |  |  |  |  |  |  |
| 10 |  |  |  |  |  |  |  |

9. When scoring is done, you may try to produce graphs using the attached Excel sheet. For this, select Table D from the left top corner until the last HS’s parameters score (thus including all scores, HS labels and parameter labels, but not the last 2 columns (HsBest10 & HsWorst0). Paste the selection into Table D in the Excel file (cell F2). Using the graphs below the table you can see if GWS match (weighted) parameter scores. If not, you may modify GWS, WF and/or parameter scores in Table D.

Finally: please answer some remaining questions below.

1. What is your area of expertise, esp. regarding broiler welfare?

10. Who are the (up to 5) most knowledgeable experts for this kind of integrated broiler-welfare assessment?

11. Could you recommend a few (up to 3) colleagues/other experts who might be willing to do this survey? If possible, email addresses are appreciated.

12. May your name be mentioned in the acknowledgements of the intended open-access publication of this study?

Yes/No

13. What is the email address I may use for the Amazon voucher?

Email:

Note: As a token of appreciation, I’m offering an Amazon voucher of 20$ for any serious and timely attempt to complete & return the survey (before May 1^st^ 2018); 30$ for any properly selected and scored set of HSs & parameters returned; 50$ for the 10 most valued returns. But note that the decision regarding the allocation of vouchers cannot be negotiated.

14. May I contact you again in the future, e.g. for clarification or notification of the publication? Note, I may be sending a reminder near the deadline unless you have indicated otherwise.

Yes/No

15. Personal communications and/or other comments/suggestions?

"Many thanks for your consideration.

For questions: email or phone: +31 317 480558. Please return your answers to [marc.bracke@wur.nl](mailto:marc.bracke@wur.nl).

### Invitation

Dear [Expert’s name],

I’m conducting a broiler welfare survey funded by the [Open Philanthropy Project](https://www.openphilanthropy.org/). I’m a scientist of Wageningen Livestock Research with a background in so-called [semantic modelling](http://marcbracke.nl/principles-of-semantic-modelling-and-risk-assessment/), requesting to share your views on integrated welfare assessment of chickens kept for meat production at the housing and management ‘systems’-level. The survey involves providing overall welfare scores for 5-6 housing systems/farms, weighting factors and scores for 4-5 welfare parameters, thus tentatively expressing what kind of welfare improvement may be expected from adopting specified alternative systems. Your answers will be kept strictly confidential. The survey has been prepared with the help of 4 (anonymous) broiler welfare scientists. They also assisted in selecting potentially suitable experts like yourself. I hope you will find this survey enjoyable/challenging, but, as a token of appreciation, I can offer a moderate Amazon voucher for timely completion of the survey (deadline May 1^st^).

If you’re not able to participate, perhaps you may be as kind as to help identify potential respondents.

Are you willing to have a look at the survey and try to provide scores?

Yes / No, thanks

You can answer the survey in Microsoft Excel (preferred), or in Email/Word. Are you willing to try using Excel?

Yes / Preferably not

Who are the (up to 5) most knowledgeable experts (in the world) for integrated broiler-welfare assessment?

Could you recommend a few (up to 3) colleagues or other experts (scientists/vets) who might be interested in answering this survey? If possible, email addresses are appreciated.

Do you wish to be notified when the study is published? We’re aiming for an open-access publication.

Yes/No

Many thanks for your consideration.

Kind regards,

Marc Bracke

**Message after acceptance:**

Dear [Name],

[That’s great/appreciated!]

Please find the survey below and attached as Word/Excel file. [If possible, please use the Excel version.] If you need help, my contact details are: [marc.bracke@wur.nl](mailto:marc.bracke@wur.nl) or phone: +31 317 480558.

As indicated, you are requested to share your views on integrated welfare assessment of chickens kept for meat production at the housing and management ‘systems’-level. The survey involves providing overall welfare scores for 5-6 housing systems/farms, weighting factors and scores for 4-5 welfare parameters (all on a scale from 0 to 10), thus tentatively expressing what kind of welfare improvement may be expected from adopting specified alternative systems. Your answers will be kept strictly confidential. Amazon vouchers are available (20-50$ for timely completed surveys; deadline: May 1^st^).

Kind regards,

Marc

### Pilot interviews

The project started with several pilot interviews. Having developed the semantic-modelling methodology, the first author (MB) first explored the required expert scoring on himself in a Microsoft-Excel worksheet. Since that appeared to work well, the intention was to construct an expert survey by email. However, when subsequently tested on a broiler-welfare expert, it appeared necessary to provide guidance to the respondents in order to enhance the likelihood eliciting the required scores. So it was decided to work towards a survey where experts would be interviewed orally (e.g. using Skype). This appeared to work well with the next expert, i.e. it took relatively little time (~1 hour) to elicit a full set of scores and the expert was enthusiastic about the approach. However, the third expert raised several objections and finally refrained from providing welfare scores. In particular, the expert objected, that for broilers, as opposed to laying hens, it was not possible to specify housing systems, which this expert appeared to associate rather firmly with housing/hardware properties. The expert also argued that welfare assessment should be based exclusively on outcomes, that ‘Backyard flock’, which was tentatively suggested as a possibly high-welfare system, was a non-production system, and that ‘Battery cage’, a possibly low-welfare system, had been superseded by modern cages with major welfare benefits such as on-farm hatching and automated harvesting (using the conveyor belt rather than catching). Though unable to provide welfare scores, this expert was most helpful in outlining variation in existing housing conditions, in suggesting potential respondents and, through raising concerns, in shaping the current survey, which was sent by email with an explicit notification that assistance (by phone/skype/email) would be available upon request (though hardly any experts made use of this offer in the end).

### Listing housing systems and parameters

A semantic-modelling approach was used to systematically describe broiler housing systems (HSs) based on statements derived from the literature. The approach requires the description of a set of HSs covering the full range of the assessment domain (i.e. the range/scope that the model is supposed to cover), namely from the least welfare-friendly but nevertheless (economically) practical/feasible HS (e.g. broiler production in traditional battery cages) to a highly ‘luxurious’, niche market or even private, broiler production ‘system’, where the latter could be chickens kept for meat as a by-product of e.g. recreational rearing or harvesting in (semi-)natural conditions. In line with this, the experts participating in the survey were asked to select their own set of HSs and assign given welfare scores (GWS), provided the HSs together covered the whole scale (from 0 to 10), thus including both a HS with a very low given welfare score (GWS) and a system with a very high GWS. In this way, the experts were free to decide for themselves which systems covered the welfare scale. They were also free to decide which parameters contributed to GWS. For proper benchmarking it was furthermore requested that experts included the most prevalent HSs (i.e. ‘conventional’) as well as the most feasible alternative HSs, as a main underlying objective was to support decision making on how to best improve the welfare of conventional broilers. Finally, to support benchmarking, experts were requested to select HSs that were described in the literature, relatively well-known among welfare experts, that preferably had typical/characteristic and unique properties (compared to the other HSs used as benchmarks), and to focus on average/typical examples of HSs, because that would reduce potential ambiguity and misinterpretation of the results.

For the questionnaire we used a predefined set of parameters derived from a systematic comparison of existing lists of parameters defined in earlier semantic models for the overall welfare assessment of different species of farm animals in relation to their housing-and-management system (including SOWEL for pregnant sows, FOWEL for laying hens, COWEL for dairy cattle and SWIM for farmed Atlantic salmon [5, 9, 10, 12]).

In order to attribute parameter levels scores (PLS) to HS, i.e. describe HSs in terms of welfare-relevant properties by assigning a parameter level score (PLS, scale 0, worst, to 10, best) the objective was to cover the whole scale of the assessment domain. Ideally, parameters should overlap as a little as possible, affect welfare more or less independently (other things being equal), and be based on scientific knowledge, i.e. an if-then rule linking welfare input and output variables supported by empirical, scientific research [5].
