# Supplementary material for: Broiler welfare trade-off: A semi-quantitative welfare assessment for optimised welfare improvement based on an expert survey
Source: PLoS One. 2019 Oct 1;14(10):e0222955. doi: 10.1371/journal.pone.0222955 (PMC6772121; doi:10.1371/journal.pone.0222955)
Supplement: S2 File — (DOCX) [file pone.0222955.s002.docx]

## S2 File. Data exploration

#### Methods

This section describes the results of data exploration, largely conduced before doing the statistical analysis reported in the main body of the paper. We describe here more details about the experts, given welfare scores (GWS) for housing systems (HS), weighting factors (WF) attributed to main welfare parameters, and parameter level scores (PLS) used to explain (together with the WF) the experts’ GWS values. We also examined possible discrepancies between GWS and calculated welfare scores (using PLS with or without WF), both between HSs and experts.

Pairwise TTests (parametric) and Sign tests (non-parametric) were done two-tailed for GWS, WF, PLS and differences between GWS and (un)weighted overall scores calculated from component (PLS, WF) scores using Microsoft Excel functions TTEST([Array 1], [Array 2],2,1) and 2*BINOMDIST([lower nr],[total number of pairs minus ties], 0.5,TRUE) respectively. For these tests we included the scores of the total of n=27 expert respondents that provided scores, and only those HSs, parameters and parameter levels had been scored least twice (n>=2). It should be noted here explicitly again that these were exploratory statistics and that where we report their ‘significant’ differences below, these are all to be regarded as tentative only (and Wilcoxon Signed Rank tests may have been more appropriate, but likely generating results ‘in-between’ the T-tests and Sign tests).

Internal consistency was checked for the experts, both at the individual expert level and at the group level, by calculating overall welfare scores from PLS and WF (both weighted and unweighted) using Pearson correlation coefficients.

As a measure of discrepancy between HS, the differences were calculated between GWS and (weighted) calculated PLS averages. Average absolute differences (after the sign was removed, i.e. negative values were turned into positive values) were (again) compared using pairwise TTests. This was done to compare HSs, i.e. to examine whether some HS’s overall welfare scores were better explained by the component scores than other HSs.

#### Experts

An invitation to participate in the survey was sent April 6 2018 to 35 experts. Even if they could not participate, these experts could recommend other experts (up to 5 top experts and up to 3 colleagues), who were then invited as well. In total 79 invitations were sent. The closing date of receiving responses was May 4, 2018.

The initial 35 experts had the following nationalities: 21 EU (of which 5 UK, 4 NL, 3 SE, 2 FR, 2 BE, 2 DE, 1 FI, 1 NO and 1 PO), 8 US, 3 AU and 3 CA. Of these, 5 experts (UK, DE, BE, US, CA) were ‘not available’, e.g. they were retired, out of office or an email failure delivery notification was received. In addition, two of the 44 recommended experts were ‘not available’ (UK, DK). Of the remaining 72 experts, 38 were willing to have a look at the survey for scoring (53%). However, in total only 27 respondents completed the survey (response rate: 38% of those invited and ‘available’; 71% of the experts who received the survey). Of the 27 respondents that provided welfare scores 13 experts did not provide any PL description (in keywords), of which 2 didn’t provide PLS either (but they did provide GWS and WF). Twenty-five experts provided WF and PLS scores to explain their GWS scores.

As regards the question (in the invitation and the survey) whether the expert wanted to be notified of the open-access publication, only 1 expert stated ‘no’; 25 answered affirmatively.

The top 5 most recommended experts were mentioned by between 15 and 8 colleagues each. Four of these were contacted and provided input as key experts in the preliminary phase of the project. Two of them did not provide scores. In the set of 27 respondents who did provide scores 1 was recommended by 4 others; 2 by 3 others, and 4 by 2 others. Twenty-two respondents stated their expertise, mostly referring to animal welfare and various research subjects (e.g. welfare indicators, lameness, social behaviour, light, contact dermatitis, health, enrichment, free range, organic); some to veterinary practice or welfare auditing (n=3). Seven of 27 respondents had a veterinary background; 22 had a background in science; 18 respondents were women; 9 were men. By continent: 19 from Europe, 6 from America (North and South), 2 from Australasia. Only 2 US experts provided PLS and GWS scores, which was not enough to analyse country as a factor in the REML analyses. Therefore, we tested by Region (EU/Non-EU).

Sixteen respondents made some comments about the difficulty of the survey. It was even described as ‘daunting’. Few respondents were (very) positive about the survey itself, e.g. one expert simply stated ‘Clear structured survey’. In the invitation the first author (MB) explicitly offered help (by phone, mail, or skype) and generally the assistance helped solve the obstacle preventing scoring (though it was infrequently requested).

#### Welfare scores for HSs

For data exploration purposes it was decided to discard all HSs that were scored only once by an expert (n=7 HSs; 3 battery-cage type systems; 2 conventional plus systems and 1 retailer scheme specific to one region in Europe).

The significance levels resulting from the Sign tests and T-tests are shown in S2 Tables A (a and b) and B.

##### S2 Table Aa. Results of pairwise comparisons of given welfare scores (GWS) for broiler housing systems (HS) as compared using Sign tests.

Note: Significance levels of pairwise Sign tests of housing systems have been sorted by overall average GWS. Systems with different superscripts differ significantly. Numbers in the HS matrix represent the 2^nd^ decimal of the p values (i.e. p=0.05 is shows as 5, so values from 5 to 9 represent trends). Green cells means the difference is not significant (p < 0.5 in the Sign test) compared to the HS linked to the grey cell. Cells with borders have n<5 (so cannot be significant in the Sign test, see the table below). BC=BattC=Battery cage, etc. ## means 'highly insignificant' (either p=1, or 'reversed number of signs compared to the rank based on overall average').

##### S2 Table Ab. Number of pairs involved in S2 Table A. (Note that n<5 cannot be significant in the Sign test.)

Orange: low numbers (<4); Blue: >10.

##### S2 Table B. Exploratory statistics and results of paired TTests of (all) GWS scores given by the experts (n=27 in total) for different housing systems (HS) for broilers. HS have been sorted by overall arithmetic average GWS per HS (thus ‘Rank’ is based on ‘Average’).

StDev: Standard deviation; Min: minimum value; Max: maximum value; Count: number of GWS scores. Significance: Systems with different superscripts ‘differ’. Characters followed by * indicate a trend difference, (p<0.1 but >0.05, corresponding to lighter green cells; green: not significant and no trend (p>0.1).

Note: TTest p values shown in the HS matrix below the row labelled ‘significance’ are pairwise comparisons of the HS corresponding to the grey cell and the system that matches the row in which the p value is reported. The first TTest compares the HS with rank 14 (Battery Cage) to the HS next in rank, i.e. rank 13, Modern Cage. The difference is not significant (p=0.2155). The next p value compared Battery cage to the next in rank (i.e. rank 12, Conventional US). This difference is significant (p=0.0019).

The number of pairs per TTest ranges from 3 (for Modern cage compared to Organic US; 7 times n=4 (of which 3 for Modern Cage)) to n=20 (for Conventional EU compared to Flock).

Note: All skew values >1 and <-1, marked darker yellow, were related to exactly 1 outlier value within the set of scores for that HS, and they were not related to any particular expert.

Fig A below shows individual expert GWS scores using primary data where each line/dot is one expert (but note that overlapping points and lines may not be visible).:

##### **S2 Fig A. Expert given welfare scores (GWS) for housing systems (HS).**


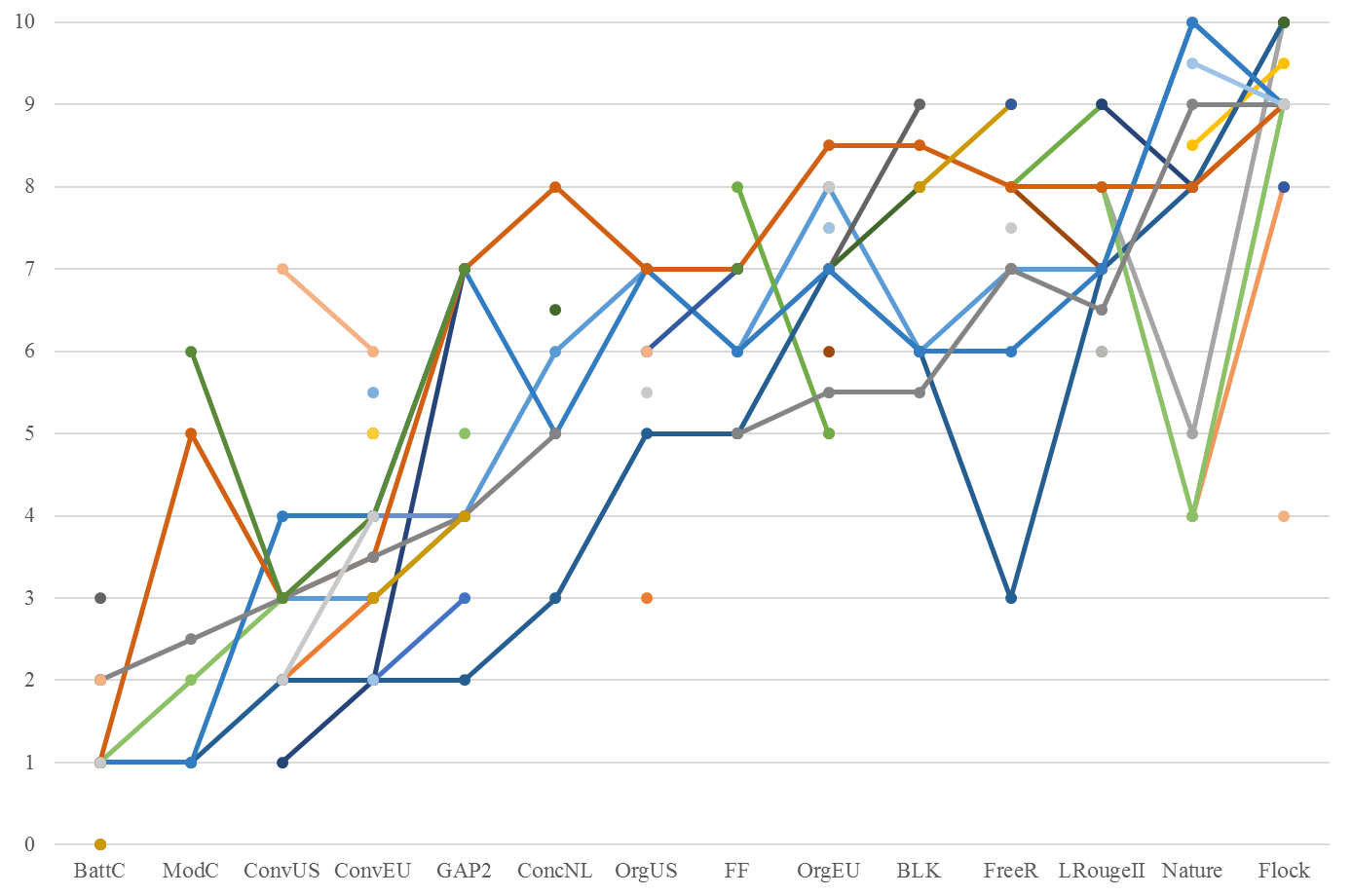


Note: This graphs shows that by-and-large the expert GWS scores correlate with the overall average, i.e. there do not seem to be distinguishable groups of experts, which think differently about welfare. For Nature there appears to be ambivalence between extremely good and inadequate, as there is incidentally (n=1) for Free range EU.

All experts who scored Battery Cage (n=16) gave this system the lowest score; all other experts gave their lowest score to either Conventional US or Conventional EU. Flock was selected as the best ‘system’ by 18 experts as the best system. Three experts gave the highest score to Nature. Organic EU was the most often scored alternative system compared to conventional (n=18). With an average score of 7.0 it had a substantially higher average welfare score than Conventional EU (3.7, n=25) and Conventional US (2.9, n=13). Of the alternative systems GAP 2 made the smallest welfare improvement (average: 4.9, n=11) and Label Rouge II be biggest (7.4, n=12) (see Table B).

The set of HSs contained several sets of related systems:

*Cages (Battery cage – Modern cage)*: Five experts gave GWS for both cage systems (Battery cage and Modern cage). Three of these gave a lower score for Battery cage; the other two experts gave both systems the same score.

Note: Four out of 6 experts that provided scores for Modern cage gave this system a lower score than any of the conventional systems. The other 2 experts gave higher scores for the Modern cage system (between 1.5 and 3 points higher scores on the scale from 0 to 10). Modern cage has the highest *variation* in scores (highest standard deviation). Note that the second highest variation in GWS scores is found on the other end of the welfare scale for Nature. To this system 3 experts gave rather low scores (4 or 5), whereas all others (10 out of 13 experts in total) gave high scores (>=8).

*Conventional (US - EU)*: Conventional EU was scored most frequently (n=25); Conventional US much less frequently (n=13). Eleven experts scored both conventional systems. Only 1 of these gave a higher score to the US system (and this was a US expert); 3 experts gave the same score (all EU experts); 7 experts gave a higher score for Conventional EU compared to Conventional US (and 3 out of these 7 were EU experts). In the Sign test this difference was a statistical trend (p=0.07) for Conventional EU being overall better for welfare than Conventional US. In the (less reliable) TTtest this difference was significant. Note that in the TTest both conventional systems (and Battery cage) differ significantly from all other systems, except from Modern cage.

*Organic (US – EU)*: Six out of 7 experts gave a higher welfare score for Organic EU compared to Organic US (and 1 expert gave the same score). This was significant in the Sign test. Four experts gave a score that was at least 2 points higher (on the 0-10 scale). The overall difference was 1.2 points (average scores of 7.0 (n=18) and 5.8 (n=8) for Organic EU and US respectively; and the difference was significant in the pairwise TTest).

*Very extensive (Flock - Nature)*: 11 experts scored both HSs. The Sign test was not significant; the TTest showed a trend (p = 0.051). Three experts have a lower score for Flock (all 3 EU experts, each giving a GWS of 9 for Flock); 7 experts gave a higher score for Flock (including 3 experts giving a much higher score: 4 or 5 points difference, i.e. these 3 experts gave a GWS < 5.5 to Nature, which may be considered ‘unacceptable’[35, 40]. This also explains the relatively high standard deviation for Nature (StDev = 2.07, n=13). Systems with a considerable range of 6 points are Nature and Flock (both ranging from 4-10, with Flock having a higher Skew value due to only one 4 outlier-score (-3.07 and -0.97 for Flock and Nature respectively; Nature had 2 scores of 4 and 1 score of 5). Conventional US also had a 6-point range (range of GWS: 1-7).

*NGO schemes (Freedom Food – Better Life)*: The difference between Freedom Food and Better Life was not significant in the Sign test, and showed a trend in the TTest (p = 0.055). Only 6 experts scored both systems: 4 experts gave a higher score to Better Life, the other 2 experts gave the same score.

*Retailer-originated schemes (Concepts NL – GAP 2):* These HSs were in the lower-welfare range of feasible alternative HSs. Concepts NL was significantly better than Conventional EU/US in the Sign test, while GAP 2 was significantly better than Conventional EU, but a trend only compared to Conventional US (p=0.06). These differences were significant in the TTest. GAP 2 scored significantly lower than Label Rouge II in the Sign test and a trend for Free Range EU (p=0.07). Concepts NL scored significantly lower than Organic EU in the Sign test, and a trend to Better Life (p=0.06).

*EU legislated (Organic EU – Free range EU – Label Rouge II)*: These systems were in the higher range of the feasible alternative systems and they did not differ in the Sign or T-test, and the average scores were similar (7.4 for Label Rouge II, 7.2 for Free range EU and 7.0 for Organic EU).

*Dutch systems (Concepts NL – Better Life)*: These systems showed a trend difference in both the Sign test (p = 0.06) and the TTest (p = 0.099, n=6 pairs). Overall, Better Life scored higher (Better Life: average 7.2, n=9; Concepts NL: average: 5.6, n=6).

##### S2 Table C. Exploratory statistics of given welfare scores (GWS) for housing systems (HS) sorted by GWS. For this table only HSs are included that have been scored by more than 1 expert, and only GWS values have been used for which also PLS have been given. Thus average scores differ from the averages presented in the previous Table. StDev: Standard deviation; Min: minimum value; Max: maximum value; Count: number of GWS scores involved.

| **HsLabel** | **Average GWS** | **Count** | **StDev** | **Min** | **Max** |
| --- | --- | --- | --- | --- | --- |
| Battery cage* | 1.36 | 14 | 0.84 | 0.00 | 3.00 |
| Conventional US* | 2.91 | 11 | 1.58 | 1.00 | 7.00 |
| Modern cage | 3.17 | 3 | 2.57 | 1.00 | 6.00 |
| Conventional EU* | 3.67 | 24 | 1.12 | 2.00 | 6.00 |
| GAP 2 | 5.00 | 8 | 1.69 | 3.00 | 7.00 |
| Concepts NL | 5.63 | 4 | 0.75 | 5.00 | 6.50 |
| Organic US* | 5.64 | 7 | 1.38 | 3.00 | 7.00 |
| Freedom Food | 6.18 | 11 | 0.98 | 5.00 | 8.00 |
| Organic EU* | 6.91 | 17 | 0.99 | 5.00 | 8.00 |
| Better Life | 7.21 | 7 | 1.35 | 5.50 | 9.00 |
| Label Rouge II | 7.28 | 9 | 1.15 | 6.00 | 9.00 |
| Free range EU | 7.50 | 13 | 0.87 | 6.00 | 9.00 |
| Nature | 8.00 | 10 | 2.01 | 4.00 | 10.00 |
| Flock* | 8.76 | 19 | 1.29 | 4.00 | 10.00 |

Note that S2 Table C is based only on GWS scores of HSs for which PLS have been provided. Based on this morel limited set of ‘verified’ scores, Modern cage has a slightly higher average GWS than Conventional US (but a high StDev, so the difference is not significant). This is one of two rank order swaps compared to Table B (where all GWS scores were included, rather than only those supported by PLS). The other swap is Label Rouge II which was the best alternative when all scores were included (S2 Table B), but second to Free range EU when only PLS-supported scores are included (S2 Table C).

As to the question which potentially feasible alternative systems could be advocated to improve broiler welfare compared to conventional housing, and what would be the ‘degree’ of welfare benefit, the experts gave higher overall welfare scores to Modern cage, which may be an improvement in the US (same average score in Table B (2.92), but 0.26 higher S2 Table C), GAP 2 (1.23 welfare points higher compared to Conventional EU; 1.99 compared to Conventional US, S2 Table B), Concepts NL, Organic US, Freedom Food, Organic EU, Better Life, Free range EU and Label Rouge II (3.72 higher score compared to Conventional EU and 4.45 higher compared to Conventional US). Also, when the Conventional EU system were adopted in the US, that could possibly generate a welfare benefit of 0.74 welfare points (S2 Table B).

Organic is not the most preferred welfare alternative among welfare experts. Other potentially feasible alternative systems (excluding Nature and Flock) have (somewhat but not significantly) higher scores: Freedom Food (compared to Organic US only); Better Life, Label Rouge II and Free Range EU (compared to both Organic US and Organic EU).

#### Parameter weighting

Two newly added parameters were left out as n=1 for each (Thermocontrol: WF=10; Contact dermatitis: WF=7).

##### S2 Table Da. Results of pairwise comparisons of weighting factors (WF) for welfare parameters as compared using pairwise Sign tests.

Parameters have been sorted by overall average WF. Parameters with different superscripts differ significantly. Numbers in the HS matrix represent the 2^nd^ decimal of the p values (i.e. p=0.05 is shows as 5, so values from 5 to 9 represent trends). Green cells means the difference is not significant (p < 0.5 in the Sign test) compared to the parameter linked to the grey cell. Cells with borders have n<5 (so cannot be significant in the Sign test). Fw=Find fd&water; La=Lameness, etc. ## means 'highly insignificant'

S2 Table Db shows the number of pairs involved in each pairwise comparison (n<5 cannot be significant in the Sign test).

##### S2 Table Db. Number of pairs involved in pairwise comparisons of weighting factors (WF) for welfare parameters as compared using pairwise Sign tests (S2 Table Da).

Orange: low numbers (<4); Blue: >10.

##### S2 Table E. Exploratory statistics and results of paired TTests of (all) WF scores given by experts (n=27 in total) for the parameters. Parameters have been sorted by overall average WF (thus ‘Rank’ is based on ‘Average’).

StDev: Standard deviation; Min: minimum value; Max: maximum value; Count: number of WF scores. TTest p values shown in the matrix below the row labelled ‘Significance’ are pairwise comparisons of the parameters corresponding to the grey cell and the system that matches the row in which the p value is reported.

S2 Fig B below shows individual expert WF scores (primary data; each line/dot is one expert; which may partially overlap).

##### **S2 Fig B. Expert weighting factor (WF) scores for welfare parameters.**


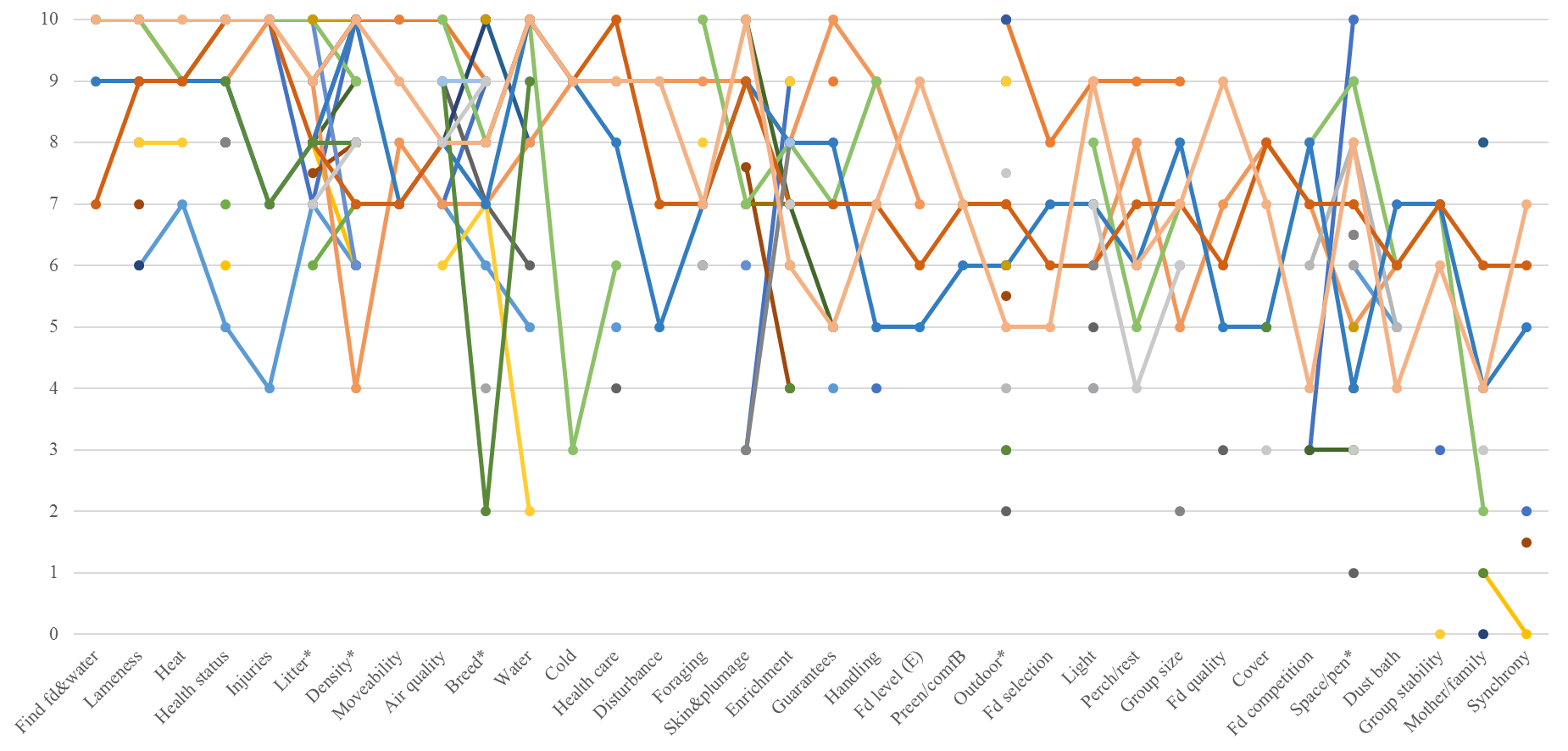


Note: This graphs shows that by-and-large the expert WF scores correlate less with the group average than the GWS scores do, and that the WF scores show more variance than the GWS scores (esp. Space/pen and Outdoor, but also Breed, Water and Mother/family). There do not seem to be distinguishable groups of experts, which think differently about welfare.

TTest p values shown in the matrix of S2 Table D are the results of pairwise comparisons of the parameter corresponding to the grey cell and the system that matches the row in which the p value is reported. The first TTest compares the parameter with rank 1 (Find fd&water) to the parameter next in rank, i.e. rank 2, Lameness. The difference is not significant (p=0.37).

The number of pairs per TTest/Sign test ranges from 3 (for all combinations with Preen/Comfb) to 23 (for Breed-Density). Note that in a 2-sided Sign test n<5 cannot be significant (min p = 0.125 when n=4, all with same sign).

Note: All skew values >1 and <-1 in Table E, marked darker yellow, were related to exactly 1 outlier value within the set of scores for that parameter, and they were not related to any particular expert.

On average WFs were high, i.e. nearly all parameters received scores >5.5 (cut-off for acceptability; scale 0-10). Only 3 parameters had scores < 5.5: Synchrony, Mother/family, Group stability. Also relatively low WF scores were given to Dust bath, Space/pen, Fd competition and Cover (all <6).

The low average score (and high variance) of Space/pen may perhaps be related to large barns in conventional systems.

High scoring and frequently scored parameters were: Find fd&water (average=9.2; n=5), Lameness (8.78; n=18), Heat (8.71; n=7), Health status (8.73. n=16), Injuries (8.50, n=8) and Litter (8.34, n=22).

Relatively frequently scored, but unimportant parameters were: Mother/family (avg 3.70; n=10), Space/pen (5.62, n=21; this parameter was listed on top of the list), Outdoor* (6.57, n=23) and Light (6.46, n=13). Note that Mother/family had a relatively large standard deviation (2.87) and range (0-8), as did Outdoor (2.27; 2-10). Breed had a large range too (2-10), but it had a high WF (average 8.0).

##### S2 Table F. Exploratory statistics of weighting factors (WF) of welfare parameters sorted by WF (total of 27 experts). For this table only parameters are included for which also PLS have been given. StDev: Standard deviation; Min: minimum value; Max: maximum value; Count: number of WF scores involved.

Most frequently selected parameters for overall welfare assessment using PLS were: Density* (n=23), Litter*, Breed*, Outdoor* (all n=20), Enrichment (n=17), Space/pen (n=16), Lameness (n=14), Health status (n=11) and Air quality (n=12), Light (n=9) and Skin&plumage (n=8).

Though not selected most often, the animal-based, output parameters Lameness and Health status had a higher average WF than the other (more often) selected main input parameters.

Most frequently selected parameters for PLS also have high WF, except Enrichment, Outdoor and Space/pen (these had average WFs of 6.9, 6.6 and 5.6 respectively with n=17, 20 and 16 experts respectively).

Parameters with a relatively high standard deviation were: Mother/family (3.6, range: 1-8, n=3), Space/pen (2.49, range 1-10, n=16) and Injuries (4.24, but here n was only 2; but note that Injuries was scored by 8 experts in total while only used twice for explaining GWS by providing PLS scores). A high standard deviation and range may indicated divergence in expert opinion, i.e. different opinions/concepts among experts.

#### PLS

##### S2 Table G. Average parameter level scores (PLS) of welfare parameter-housing system (HS; see S2 Table A for HS abbreviations).

| **ID** | **Parameter**  **Label** | **BattC** | **Cus** | **Ceu** | **Gap2** | **Ous** | **Oeu** | **FF** | **BLK** | **LRouII** | **FreeR** | **Flo** | **Nat** | **H10** |
| --- | --- | --- | --- | --- | --- | --- | --- | --- | --- | --- | --- | --- | --- | --- |
| 1 | Space/pen* | 1.45 | 3.50 | 3.31 | 6.20 |  | 7.33 | 5.40 | 7.08 | 5.50 | 6.29 | 8.38 | 9.00 | 10.00 |
| 2 | Density* | 1.15 | 3.10 | 3.30 | 5.86 | 4.71 | 7.60 | 5.80 | 6.67 | 6.63 | 6.92 | 8.65 | 9.25 | 10.00 |
| 4 | Breed* | 2.09 | 3.44 | 2.90 | 4.29 | 4.60 | 7.04 | 6.29 | 6.00 | 7.43 | 6.89 | 8.07 | 9.38 | 10.00 |
| 5 | Litter* | 0.18 | 4.13 | 4.58 | 6.33 | 4.20 | 5.86 | 7.25 | 7.90 | 6.17 | 6.91 | 8.13 | 9.00 | 10.00 |
| 6 | Air quality | 3.40 | 5.14 | 5.00 | 6.80 | 6.80 | 7.14 | 6.67 |  |  | 7.50 | 8.44 | 9.57 | 10.00 |
| 7 | Enrichment | 0.33 | 1.50 | 1.09 |  |  | 7.42 | 6.00 |  | 6.58 | 7.95 | 8.96 | 10.00 | 10.00 |
| 8 | Outdoor* | 0.09 | 0.00 | 0.06 |  | 6.00 | 7.69 | 1.67 | 6.10 | 7.08 | 8.27 | 9.12 | 9.50 | 10.00 |
| 15 | Lameness | 2.25 |  | 3.50 |  |  | 7.22 | 6.14 |  | 6.86 | 7.71 | 7.30 | 9.20 | 10.00 |
| 16 | Skin&plumage |  |  | 3.88 |  |  |  |  |  |  | 5.83 | 7.14 |  | 10.00 |
| 30 | Light | 3.67 | 4.10 | 4.44 |  |  | 6.56 |  |  |  |  | 8.22 |  | 10.00 |
| 32 | Health status | 4.42 | 4.80 | 4.86 |  |  | 4.79 | 6.50 |  | 6.40 | 6.40 | 7.50 | 6.00 | 10.00 |

Note: This table only lists average PLS where n>4 (i.e. scored by more than 4 experts).

Average PLS do not explain the difference between Conventional US and Conventional EU. The main difference between Organic US and Organic EU is expressed in various parameters, especially Density and Breed (and to a lesser extent by Litter, Air quality and Outdoor).

Nature has higher average PLS than Flock for all but 1 parameter (only lower for Health status, which is 1 parameter out of 9; 8 out of 9 parameters have a higher score for Flock).

Battery cage had lower average PLS than Conventional US (and Conventional EU) for 8 out of 9 parameters, except for Outdoor (where score is 0.09 vs 0.00 (and which does not make sense).

##### S2 Table H. Results of comparisons of expert parameter level scores (PLS) compared per parameter between housing systems (HS) using pairwise Sign tests.

Legend: The first (upper left) green cell gives the second decimal of the Sign-test p-value comparing Battery cage (grey) to Conventional US for the parameter Space/pen; ##: not significant. The upper left orange cell gives the number of pairwise comparisons (if n<6, the Sign test cannot be significant). Green: not significant; Yellow: significant; Blue: higher number of pairs.

##### S3 Table I. Results of TTests and exploratory statistics.

S2 Fig C below gives an impression of PLS values per parameter across HSs.

##### **S2 Fig C. Parameter level scores (PLS) per parameter across HSs.**


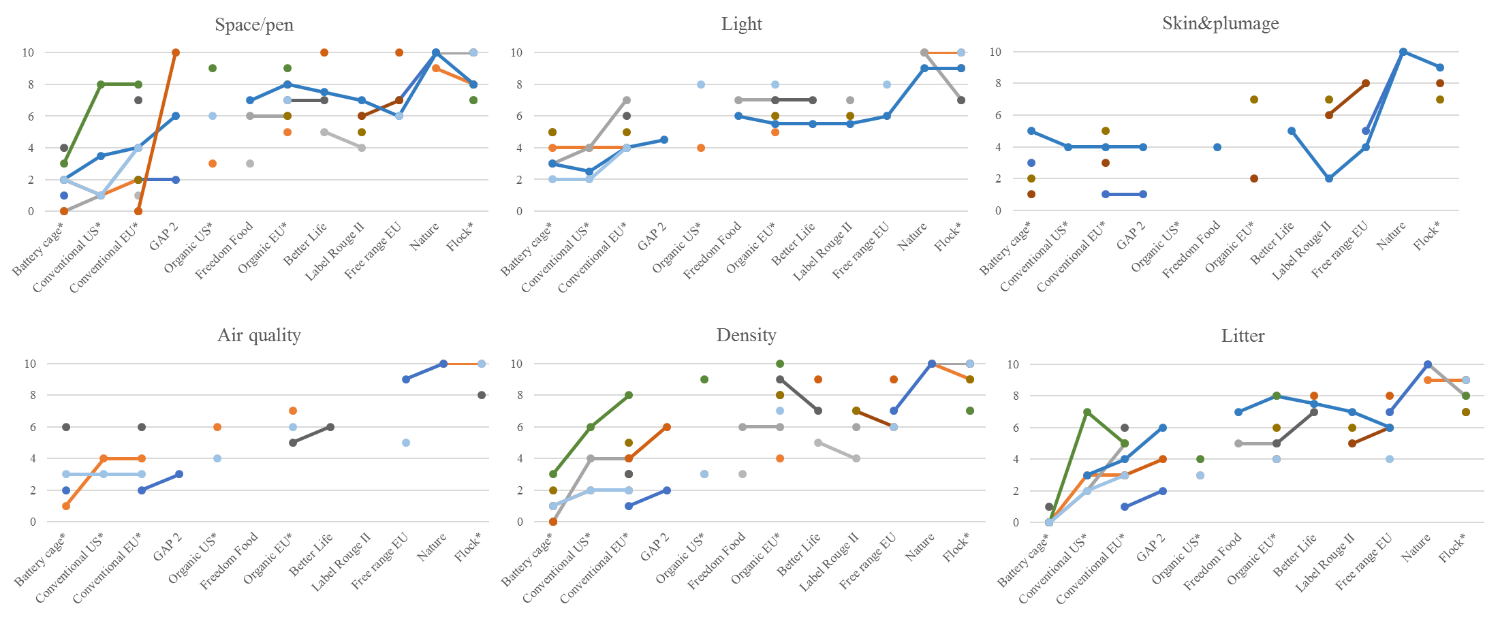


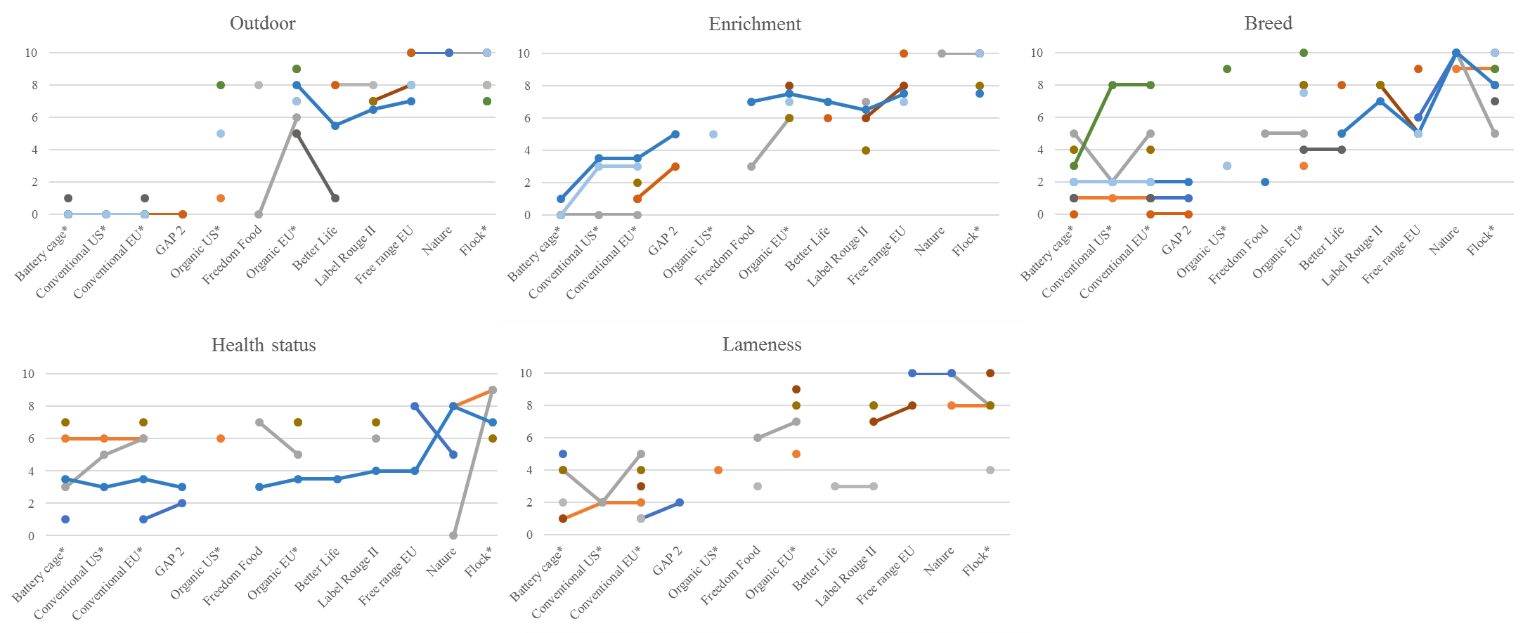


The Sign tests of PLS scores show rather few, and mostly not interesting/unsurprising, significant pairs, mainly due to the low numbers of pairs. For example, Space/pen BC<CE, OE and FL (for HS abbreviations see S2 Table A), and CE<OE, LR, NA, FL. In addition there were several trends (due to n=5, all pointing in the same direction): BC<LR, FR; CE<FF,BL; OE<FL; LR<FL. Note that the PLS value of Space/pen is not just the amount of space, but the welfare value of the given amount of space available to the birds according to the experts.

Light: BC<OE,FL; Trend: CU<FL.

Skin&plumage, Air quality and Health status: all NS (very low number of pairs with n>4).

Outdoor: BC<OE,FR*,FL; CU<FL; CE<OE,FR,NA*,FF<FL*;OE<FL;FR<FL*; *: trend.

Enrichment: BC<CE, OE,LR,FR,FL; CU<FF*,OE*,FL; CE<FF,OE,LR,FR,NA*,FL; FF<FL.

Breed: BC<OE,LR*,FR,FL; CE<OE,FR,NA*,FL.

Density: BC<CE,LR,FR,FL; CU<FL*; CE<GA*,OE,LR*,FR,FL*; OE<FL; LR<FL*.

Litter: BC<CE,OE,FR,FL; CE<OE,FR,FL; OE<FL.

Lameness: BC<OE, LR*,FL; CE<OE,NA, OE<NA.

TTests showed more significances, but still few for parameters without any significance in the Sign tests (Skin&pumage, Air quality, Health status). Most significant pairs were seen with Enrichment and Density; and also most significances for BC and CE compared to the other HSs.

In the pairwise Sign tests (taking into account only cases where n>4): The PLS for Space/pen in Battery cage did not differ significantly from Space/pen in Conventional US, despite N=5. Two out of 5 experts gave a PLS of 2 for Battery cage and 1 for Conventional US. This is counterintuitive. Battery cages provide much less space than conventional barns and it is unlikely any expert could justify that the amount of space in a Battery cage is better for welfare than the amount of enclosure space in a conventional barn. It is most likely then that here 2 out of 5 experts did not fully understand what they were supposed to be doing in assigning PLS values, or they were not being rational (e.g. wanting to give low PLS values to Battery cages in order to justify the low overall GWS score). Further exploration of the parameter Space/pen showed more apparently unexplainable scoring (e.g. an expert assigning a higher PLS to GAP 2 than to Flock and Nature; another scoring Space/pen higher for Organic US than Flock, while the same as Organic EU; a third expert gave a low score to Free range EU and a higher score to Conventional EU for Space/pen). It appeared that some respondents may have included space quality in this parameter while others didn’t. These questionable scores, in addition to the relatively low overall WF and high variance for this parameter, and the fact that conventional systems already provide a considerable amount of enclosure space, led to the decision to exclude this parameter (Space/pen) in the final statistical analyses as reported in the main body of the paper.

The figures above suggest that (PLS scores for) Air quality appears to be well correlated with overall GSW average for HSs. For Health status the correlation appears to be rather low. No (main) parameter showed a (clearly) negative correlation with overall welfare.

Very low score and very little variance seem to be present for Litter and for Enrichment in Battery cage. Outdoor appears to be an ‘all-or-nothing’ variable (yes/no), i.e. it seems to have been scored as presence (yes/no), while there could be a considerable differentiation in the quality and even in presence (e.g. for porches/veranda’s).

#### Discrepancy GWS – PLS & WF between HSs and experts

When comparing discrepancies between housing systems in the degree to which component scores (PLS and WF) could explain GWS values we found that Nature has the highest average and standard deviation of absolute discrepancy between GWS and weighted average PLS scores (of systems with n>4). Flock has the highest average of non-absolute discrepancy values. Very few significant differences in discrepancy were found between HSs. For absolute values only 1 significant difference was found in the pairwise Sign tests: Nature (average discrepancy = 1.8) – Freedom Food (average: 1.1) (p=0.03, n=6). This pair was also significant in the TTest, which also revealed 1 other significant difference: Conventional EU (average discrepancy: 1.4) – Free range EU (average: 0.84; n=12 pairs). In non-absolute comparisons 3 significant Sign tests were found, all related to Battery cage.

When comparing experts regarding discrepancies between given (GWS) and (weighted, unweighted and differentiated) calculated scores using Pearson correlation coefficients we found 2 ‘outlier’ experts (nr 4 and 79), which showed negative correlations, and one experts that showed a low correlation for weighted and unweighted scores, but a reasonable correlation with differentiated weighting (See S2 Table J). Based on this finding we decided to exclude the two negatively correlating experts from the final analyses, also because we failed to make (logical) sense of their PLS and WF scores to explain their GWS scores.

##### S2 Table J. Pearson correlation coefficients of each expert’s given welfare scores (GWS) with various ways of ‘explaining these scores by calculating overall welfare from welfare components (parameter level scores, PLS and weighting factors WF). Three main types of calculation shown here are weighted (average of PLS multiplied by WF and divided by the sum of WF, labelled PLSWF), unweighted welfare, i.e. average of PLS values where all WF=1 (labelled PLSWF1), and a differentiated welfare calculation (where WF=1 when PLS=10 (high) and PLS is weighted with the expert’s assigned WF value of the parameter when PLS is low, i.e. 0, and proportional when PLS is between 0 and 10). Experts 4 and 79 show orange because they have negative correlation coefficients. Expert 66 has low correlations for the weighted and unweighted welfare calculation, but a reasonable correlation for the differentiated calculation (R=0.78).

Further data exploration showed that expert 4 gave lower PLS to Air quality, Density*, Group size, Space/pen and Light for Flock* and Nature, than for Conventional EU/US and GAP 2. This expert seems to have misunderstood the point of explaining GWS scores using PLS scores, which were supposed to be component welfare scores on a scale from 0 worst to 10 best. This expert also gave puzzling PLS for Light (7 for Freedom Food, Better Life and Concepts NL vs. 4 for Free Range EU, Label Rouge II, Organic EU and US), as e.g. Organic US uses porches a bit similar to Better Life. Also, all PLS for Water were set at 5, thus failing to produce any explanatory value. As a result, the various PLS values attributed to each HS did not ‘add’ up to GWS in any logically conceivable way and this was showing up as a strong negative Pearson correlation (– 0.8) between GWS and calculated welfare scores. In particular, the low PLS for Nature could not explain the high GWS for this HS. When we compared provisional ALM analyses with and without the two ‘outlier’ experts we also found that the infrequently scored parameter Dustbath (n=2) had an exceptionally high Beta-coefficient, probably related to the fact that the PLS of this parameters were the main ‘factor’ that seemed to correlate with the GWS of expert 4. Since we didn’t want to make aberrant recommendations based on only a few experts and since the survey was structured to provide rationally explainable, science-based scores (e.g. by providing bar charts allowing a comparison between GWS and component PLS and WF scores), we felt justified in (nonetheless reluctantly) excluding these two experts from the main analyses. Expert 79, who was also excluded, had less negative correlations (-0.7 to -0.1), but when comparing the PLS and WF values with GWS there was, again, no logical way we could conceive of to explain GWS from component scores either.

The average Pearson’s correlation coefficient between GWS and unweighted PLS welfare calculation increased from 0.77 to 0.90 when the two outlier experts, who did not seem to have understood the task of assigning PLS, were excluded. For weighted PLS the corresponding values were 0.78 and 0.89 respectively (and 0.79 and 0.91 for differentiated weighting).

The Pearson’s correlation coefficient with GWS was 0.85 and 0.86 for unweighted and weighted calculated welfare scores (CalcWS) respectively (n=147 combinations of HS and experts; all p<0.001).

### Summary table

S2 Table K shows the average PLS scores for the main HS and WF.

##### S2 Table K. Summary table showing average values of WF (weighting factors; n=23 experts, including only experts that provided valid PLS scores, i.e. excluding the 2 outlier experts), GWS (given welfare scores; n=27 experts) and PLS (with n>=2 per PLS). Grey cells show missing values (with n<2). The housing systems (HS) Modern cage and Concepts NL have also been left out here, as these had only n=6 GWS scores per HS. The cells in the table show average PLS values and N (number of scores) per parameters and HS. For example, the average PLS of Lameness in Battery cage* is 2.25 with n=8. Space/pen* is shown in grey as this parameter showed irrational scoring and was left out of the statistical analysis.
