# Supplementary material for: Broiler welfare trade-off: A semi-quantitative welfare assessment for optimised welfare improvement based on an expert survey
Source: PLoS One. 2019 Oct 1;14(10):e0222955. doi: 10.1371/journal.pone.0222955 (PMC6772121; doi:10.1371/journal.pone.0222955)
Supplement: S3 File — (DOCX) [file pone.0222955.s003.docx]

## S3 File. Additional statistical analyses

This file describes some additional statistical analyses, esp. supplementary ALM analyses.

#### Expert concordance

Cronbach’s Alpha was provisionally calculated as a measure of the degree to which the different experts were assessing the same construct (overall welfare scores of 27 experts as represented by their GWS and WF values [31]). The calculations were provisional because in order to be able to calculate Cronbach’s Alpha missing values were substituted by average scores. Since this analysis was rather tentative, we also calculated the averages of Pearson’s correlation coefficients between all pairs of experts’ scores for both GWS and WF without data substitution.

The provisional Cronbach’s Alpha for expert GWS scores of HSs was 0.58 (n=27 experts). Cronbach’s Alpha for the expert WF scores was 0.79. The average Pearson correlation coefficient between the GWS of all pairs of experts (without substitution of missing values) was 0.80, and it was 0.28 for WF values.

#### Significant differences between housing systems and parameters in the REML analysis

S3 Table A shows significant differences between HSs based on a post-hoc test using Bonferroni correction.

##### S3 Table A. Significance levels of pairwise comparisons of given welfare scores (GWS) of housing systems (HSs). HSs are sorted by the Predicted Mean GWS of the REML analysis. The table also shows Standard Errors (StdErr) and the number of experts having scored the system (n=27 experts in total). Groups of HSs sharing a similar welfare level are identified using a similar colour-shading for HS labels. Red: significant using Bonferroni correction (p<=0.05); Green: not significant (p>0.05). HSs with different superscript characters differ significantly.

S3 Tables Ba and Bb show significant differences between pairs of parameters based on a post-hoc test with and without Bonferroni correction (S3 Table Bb shows LSD values).

##### S3 Table Ba. Significance levels (p values) of pairwise comparisons of weighing factor (WF) scores assigned by the experts (n=27) to parameters. Parameters are sorted by the Predicted Means of the REML analysis. The table also shows Standard Errors (Std. Error) and the number of experts having scored the parameter (N). Red: significant after Bonferroni correction (p<=0.05); Green: not significant (P>0.05).

##### S3 Table Bb. Significance levels (p values) of pairwise comparisons of weighing factor (WF) scores assigned by the experts (n=27) to parameters. Parameters are sorted by the Predicted Means of the REML analysis. The table also shows Standard Errors (Std. Error) and the number of experts having scored the parameter (N). Red: significance level based on Least Square Differences (LSD) (p<=0.05); Green: not significant (P>0.05).

#### ALM analyses related to weighting

The primary automatic linear modelling (ALM) analyses [32-34] on the PLS values for each parameter and expert (n=23 experts in total) were performed to see to what extent parameters could explain the (variance in) GWS values.

We also examined the contribution of weighting factors (WF) in the ALM analyses, as including only PLS would imply explaining GWS from unweighted PLS values only, while using WF would imply explaining GWS from weighted PLS. This was done by calculating PLS fractions from PLS values and WF scores.

PLS fractions combine the information provided by the parameter’s WF and PLS. They are calculated by multiplying the WF and PLS (for any given parameter, HS and respondent) and dividing this value by the sum of the WF’s for all parameters used by the respondent to explain his/her GWS. The sum of PLS fractions for all parameters selected by an expert add up to the weighted overall welfare score (scale 0-10) for any HS, which explains the GWS to the extent that the scores are similar.

In addition to the analyses using PLS fractions, several preliminary ALM regressions were performed including both PLS and WF scores of parameters (numeric scale variables) and the categorical variables System (HS), Parameter and Expert (string nominal variables) to explain GWS.

In all cases WF did not significantly contribute to the model, and WF was of low importance. Since WF did not show up as a predictor of GWS, we decided to focus on explaining GWS based on (unweighted) PLS values, and limit the presentation of the ALM analyses using (weighted) PLS fractions. The main analysis of PLS is more straightforward than using PLS fractions, and the influence of WF was also expected to be low based on what we found earlier [4, 36], and given the fact that the experts were only selecting main parameters (which of which was thus expected to be relatively important).

#### Supplementary ALM analyses explaining overall welfare from component scores

This section supplements the ALM analyses presented in the section ‘Regression analyses explaining overall welfare from component scores’.

##### All parameters and HSs

A similar analysis to the one presented in the sub-section ‘All parameters and HSs’ in the main paper, but now using PLS fractions resulted in a similar explained variance (84.1% vs 85.3%), and partly similar parameters showing up in the model as main factors (Health status, Density) together with Mother/family. The Beta-coefficient of the latter was rather high (3.1), but n for this parameter was based on only 3 experts.

##### All parameters without HSs

A similar analysis to the one presented in the sub-section ‘All parameters without HSs’ in the main paper, but now using PLS fractions resulted in a reduced explained variance (68.1% vs 80.9%), and partly similar parameters showed up in the model as main factors (Density*, Enrichment and Health status, which were significant and of relatively limited importance). However, also a number of ‘new’ parameters showed up (i.c. Perch/rest, Water and Skin/plumage), and in addition to one negative Beta-coefficient (for Water: -2.3), one parameter (Perch/rest, ) had a rather large Beta-coefficient (5.5) implying that a small change in PLS fraction would already result in a substantial increase in GWS. However, n was low for these parameters (n=3 for Water and Perch/rest; n=6 for Skin/plumage). Thus, the effect was based on the scores of a limited number of experts.

##### Input parameters and HSs

The ALM model using HSs and the PLS scores of only input parameters as variables in the model explained 84.8% of the GWS variance (Information criterion 22.6 ). Significant differences between (clusters of) HSs explained most of the variation in GWS (Importance: 0.54) with smaller, but significant contributions from the parameters Health care, Density*, Breed*, Air quality, Enrichment and Outdoor (Importance decreasing from 0.14 to 0.03; see S3 Table C; Guarantees was almost significant (p=0.050). The significant input parameters contributed to GWS between 4.5 (for Health care) to -0.8 welfare points (for Outdoor) when raising PLS from 0 to 10. Note that the Beta-coefficient for Outdoor was negative, implying that a higher PLS negatively contributed to GWS. HSs were clustered in similar groups as for the ALM on all parameters.

##### S3 Table C. Results of automatic linear modelling (ALM) regression analysis explaining given welfare scores (GWS) scores based on clustering of housing systems (HS) in welfare levels and parameter level scores (PLS) of only input parameters. Beta: Beta-coefficient; T: T-test result; Signif: Significance level (p value, see section ‘Statistics’, also for ‘Importance’); Ref: HS cluster reference (see text).

A similar analysis using PLS fractions resulted in a similar explained variance (82.2% vs 84.8%), and partly similar parameters as main factors in the model (Air quality, Density*) together with Water (Beta-coefficient of the latter was negative -1.5, but n for this parameter was based on only 3 experts).

##### Input parameters without HSs

A similar analysis to the one presented in the sub-section ‘All parameters and HSs’ in the main paper, but now using PLS fractions resulted in a reduced explained variance (62.4% vs 77.3%), and partly similar parameters as main factors in the model (Enrichment, Density*, Breed*) together with new ones (Water, Synchrony and Perch/rest; Beta-coefficients -2.6, 27.3 and 2.4 respectively, based on n= 3, 2 and 3 expert scores respectively).

##### Output parameters and HSs

The ALM model including HSs and only output parameters explained 79.9% of the GWS variance (Information criterion 55.4). Significant system differences explained most of the variation in GWS with a significant (but only moderately important) addition of the parameter Health status (see S3 Table D). A PLS of 10 rather than 0 for Health status added 3.3 to the GWS. HSs were clustered as in earlier ALM analyses.

##### S3 Table D. Results of automatic linear modelling (ALM) regression analysis explaining given welfare scores (GWS) scores based on clustering of housing systems (HS) in welfare levels and parameter level scores (PLS) of only output parameters. Beta: Beta-coefficient; T: T-test result; Signif.: Significance level (p value, see section ‘Statistics’, also for ‘Importance’); Ref: HS cluster reference (see text).

A similar analysis using PLS fractions resulted in a similar explained variance (80.5% vs 79.9%) and one (similar) parameter was significant in the model (Health status).

##### Output parameters without HSs

A similar analysis to the one presented in the sub-section ‘Output parameters without HSs’ in the main paper, but now using PLS fractions resulted in a similar explained variance (34.5% vs 39.4%), and the same parameters in the model (Lameness and Health status).

##### Input or output parameters affecting each HS separately

Finally, two ALM analyses were carried out to determine the effect of input or output parameters on each HS separately (S3 Table E). Variance in GWS of Conventional EU* was affected mainly by Breed* as the (only significant) input parameter, and by Health status as the only significant output parameter. The former contributed 2.2 welfare points to GWS, the later 3.2 points. For Conventional US* Litter* and Air quality were significant input parameters (with a very high accuracy of 93.%). Air quality contributed 2.6 welfare points, and the parameter Litter* was classified in 2 clusters such that a PLS value of 7 (reference) instead of 2, 3 or 6 resulted in an improved GWS of 2.9 points (see ALSO THE explanation below the table). The main input parameter affecting Organic US* was Outdoor* (with 2.9 welfare points improvement from PLS=0 to PLS=1). Main output parameters affecting Organic EU* were Lameness and Health status. Density* was selected for both Battery cage* (low-end welfare) and Nature and Flock* (high-end welfare).

##### S3 Table E. Significant input and output parameters related to the given welfare scores (GWS) per housing system (analysed for sets of only input and only output parameters separately, shown in the upper and lower half of the table respectively, separated by the line between Input and Output parameters in column In/Out). For these analyses the total number of experts was 23, and n>=2 expert PLS values per parameter. Beta: Beta-coefficient; T: T-test result; Signif.: Significance level (p value, see section ‘Statistics’, also for ‘Accuracy %’).

Parameters marked * were preferred for scoring in the survey. (Idem) means the same model was used as the previous row (same accuracy and intercept). Note that all negative beta-coefficients are the result of clustering by the ALM procedure similar to the clusters of HSs in previous analyses (so compared to a reference cluster for which the beta-coefficient is 0.000). This is to be interpreted such that for the row with the negative coefficient the GWS was the negative value lower than the intercept for the PLS values specified compared to the reference. For example, to improve Conventional US* Litter* is significant. At value Litter*=7 the GWS for this system is the intercept value (namely 3.2), whereas when Litter* is reduced to a PLS of either 2, 3 or 6, then GWS is 0.3 (=3.2-2.9, i.e. Intercept minus Beta-coefficient).

Note that for the analysis in S3 Table E we used n>=2 PLS scores per parameter. However, because we are most reluctant to recommend system improvements that could be based on potentially only a few expert scores, we also did these ALM analyses for n>=5 scores per parameter. The results were identical to S3 Table E, except that for Battery cage* instead of Health care (with n=3) Outdoor* was selected as a relevant variable (where a value of 0 rather than 1 would reduce the GWS by 1.5 welfare points).

#### Summary tables

S3 Table F shows a summary of the ALM analyses across HSs (also including results presented in the main body of the paper).

##### S3 Table F. Summary of ALM analyses explaining given welfare scores (GWS) based on clustering of housing systems (HSs) in welfare levels (coded as ‘&HS’ in the first column) or without HS clustering (coded as ‘no HS’) and using the (unweighted) parameter levels scores (PLS) of all parameters (coded ‘All p.’), only input parameters or only output parameters (n=23 experts in total). The table also lists the percentage of variance in GWS explained for each PLS model, the significant parameters, and their beta-coefficients. A comparison is also made to the ALM analyses using (weighted) PLS fractions (where PLS have been multiplied by the parameter’s WF and divided by the sum of WF). Similar parameters selected in the PLS fraction models are indicated by ‘y’ (yes); different parameters (from the ALM using PLS) are specified (and are unrelated to the given beta-coefficients). The second model is presented in bold as we believe that is to be regarded as the primary model to be used to improve the welfare of broilers.

Most variance in GWS was explained in the ALM model when using all available parameters and HSs (85.3%). Least variance was explained when only output parameters were included (39.4%). Understandably, ALM analyses with HSs had a higher % explained than similar models without HSs.

Health status was selected in all ALM models where this output parameter could be selected (i.e. either using all parameters or only output parameters, and both for models with and without HS clustering). Density*, Health care and Enrichment were included in all models where these input parameters could be selected. Breed* was selected in the two models with only input parameters (with and without HS clustering), and when all parameters were included together with HS cluster, but not in the model of all parameters without HS clustering. Parameters included in some, but not all models were the input parameters Litter* (all parameters without HSs), Air quality and Outdoor (only input with HSs), and the output parameter Lameness (only output, without HSs).

The ALM analyses using PLS fractions showed a similar level of explained variance, with a drop of over 10% in the model of all parameters without HS clustering, and the model of only input parameters without HS clustering. PLS fraction analyses selected partly the same parameters (esp. Health status*, Breed* and Density*, and also Enrichment, Air quality and Health care) and partly new parameters, which generally had a relatively low number of values such as Mother/family, Perch/rest, Water, Skin/Plumage and Synchrony (n=3-6).

##### S3 Table G. Core output table of the expert survey compiling the results of ALM and REML analyses on given welfare scores (GWS) of 14 housing systems (HSs), weighting factors (WF) of welfare parameters and parameter level scores (PLS). GWS: row of predicted mean GWS values for the 14 HSs using REML (sorted and colour-coded in 6 levels/clusters of welfare levels as suggested by both REML and ALM); StErr.: Standard error. N: number of GWS per HS (all REML, n=27 experts in total). Column ‘Mean WF’ sorts parameters from high to low WF. These are not significantly different from each other in REML (also n=27 experts in total). Column ‘N (ALM)’ shows the number of WF values in the ALM analyses on a more restricted dataset (, namely using only parameters that were also used for PSL scoring; (n= 23 experts in total). The table shows only parameters that were significant in ALM analyses using 3 sets of parameters (all, only input, only output parameters) to explain GWS using PLS values (and without HS clustering in welfare levels). Beta-coefficient values in bold are what we recommend as primary results to improve the welfare of broilers. The cells in the cross-table are beta-coefficient results from ALM analyses per system (S3 Table E; see also the note below the table on how to read this table). This table is a compilation of information presented in earlier tables in this publication: S3 Table A, S3 Table B and Tables 6, 7 and 8 in the section ‘Regression analyses explaining overall welfare from component (i.e. PLS) scores’.

^ The beta-coefficient result for impact of Health care on Battery cage* is based on n=3 expert scorings. The same ALM analysis with n>=5 rather than n>=2 resulted in Outdoor being significant (as specified in the table; see S3).

Note: The column ‘Beta-coefficient’ shows the effect that applies across housing systems depending on the set of parameters selected for welfare prediction specified in the preceding column (labelled ‘Set of parameters used in ALM’). Beta-coefficients specify the impact on GWS per unit of change in PLS, so improving PLS from 0 to 10 for a beta-coefficient value of 0.64 for Lameness (provided this range has been assigned in the dataset) has an impact of 6.4 GWS welfare points in case only output parameters are used to explain GWS. Note that the corresponding beta-coefficient for Health status is 0.55, but only 0.38 when all parameters are included in the ALM model (both without HS clustering). The latter is primary, and thus shown in bold. The beta-coefficient of Health care for ‘All’ (0.40) is not shown in bold, because n is low (namely 3). The body of the cross-table also shows beta-coefficient results of AML analyses per HS, using only the set of input or the set of output parameters (not all). A positive beta-coefficient value in the cross-table between 0 and 1 is to be interpreted as explained for the column ‘Beta-coefficient’. Negative values such as -1.2 in ‘-1.2if0vs1/2/3’ for Density* in relation to Battery cage* implies that a PLS value of 0 rather than 1, 2 or 3 reduced GWS by 1.2 points. A potentially noteworthy finding of the ALM analyses per system was the high accuracy (93.4%) of explaining GWS of Conventional US* by (PLS of) Litter* and Air quality.
